# Serum *N*-Glycan Changes in Rats Chronically Exposed to Glyphosate-Based Herbicides

**DOI:** 10.3390/biom14091077

**Published:** 2024-08-28

**Authors:** Moyinoluwa Adeniyi, Cristian D. Gutierrez Reyes, Jesús Chávez-Reyes, Bruno A. Marichal-Cancino, Joy Solomon, Mojibola Fowowe, Sherifdeen Onigbinde, Jorge A. Flores-Rodriguez, Md Mostofa Al Amin Bhuiyan, Yehia Mechref

**Affiliations:** 1 Department of Chemistry and Biochemistry, Texas Tech University, Lubbock, TX 79409-1061, USA; moadeniy@ttu.edu (M.A.); cristian.d.gutierrez-reyes@ttu.edu (C.D.G.R.); joy.solomon@ttu.edu (J.S.); mfowowe@ttu.edu (M.F.); sonigbin@ttu.edu (S.O.); mdmobhui@ttu.edu (M.M.A.A.B.); 2 Center of Basic Sciences, Department of Physiology and Pharmacology, Universidad Autónoma de Aguascalientes, Aguascalientes CP 20131, Mexico; jesus.chavezr@edu.uaa.mx (J.C.-R.); bruno.marichal@edu.uaa.mx (B.A.M.-C.); jorge12_flores@hotmail.com (J.A.F.-R.)

**Keywords:** glyphosate, glyphosate-based herbicide, neurotoxicity, *N*-glycans, LC-MS/MS

## Abstract

Glyphosate, the active ingredient in many herbicides, has been widely used in agriculture since the 1970s. Despite initial beliefs in its safety for humans and animals due to the absence of the shikimate pathway, recent studies have raised concerns about its potential health effects. This study aimed to identify glycomic changes indicative of glyphosate-induced toxicity. Specifically, the study focused on profiling *N*-glycosylation, a protein post-translational modification increasingly recognized for its involvement in various disorders, including neurological conditions. A comprehensive analysis of rat serum *N*-glycomics following chronic exposure to glyphosate-based herbicides (GBH) was conducted using liquid chromatography-tandem mass spectrometry (LC-MS/MS). The results revealed significant changes in the *N*-glycan profile, particularly in sialylated and sialofucosylated *N*-glycans. The analysis of *N*-glycans across gender subgroups provided insights into gender-specific responses to GBH exposure, with the male rats exhibiting a higher susceptibility to these *N*-glycan changes compared to females. The validation of significantly altered *N*-glycans using parallel reaction monitoring (PRM) confirmed their expression patterns. This study provides novel insights into the impact of chronic GBH exposure on serum *N*-glycan composition, with implications for assessing glyphosate toxicity and its potential neurological implications.

## 1. Introduction

Glyphosate [N-(phosphonomethyl) glycine] was initially identified in 1950 by Dr. Henri Martin [1]. However, it was not until 1970 that Dr. John Franz identified its herbicidal function, and it was first formulated and sold as a commercial herbicide by Monsanto in 1974 [2]. Since then, glyphosate has been widely utilized in modern agriculture across the globe. Glyphosate-based herbicides (GBHs) incorporate glyphosate as the primary active constituent. The herbicidal action of glyphosate is exhibited by inhibiting the shikimate pathway, a vital biochemical process responsible for synthesizing aromatic amino acids in plants, fungi, and certain microorganisms [3]. Glyphosate specifically inhibits the enzyme enolpyruvylshikimate-3-phosphate synthase, a penultimate step in the shikimate pathway resulting in the blockage of expression of essential amino acids such as tyrosine, phenylalanine, and tryptophan [4], thereby causing the termination of the target organism within a few days [5].

The absence of the shikimate pathway in humans and animals led to the conclusion that glyphosate presents no adverse health effects to humans or animals. However, the consideration of potential limitations on the utilization of glyphosate arose with the publication of initial studies that unveiled its detrimental impact on the health of humans and its capacity to accumulate in soil, water, and food items [6]. Following this revelation, there has been growing interest in glyphosate-induced toxicity for decades [7]. As of 2015, the European Food Safety Authority (EFSA) and the International Agency for Research on Cancer (IARC) have an opposing stand on glyphosate and its potential to cause cancer [6]. EFSA categorized glyphosate as a chemical with unlikely carcinogenic potential, while (IARC) classifies it as a probable carcinogen [6,8,9]. However, several in vitro and in vivo studies have reported neurotoxic effects of glyphosate in mammals [10]. This makes it necessary to identify potential biomarkers indicative of glyphosate toxicity in mammals.

*N*-linked protein glycosylation is the most abundant and extensively studied form of protein glycosylation [11]. The intricate process of *N*-glycosylation involves the covalent attachment of glycans/oligosaccharides to asparagine residues within the consensus sequence Asn-X-Ser/Thr, where X represents any amino acid except proline. This structured modification plays a pivotal role in intricately regulating cellular processes influencing protein folding [12], cell-to-cell communication [13], recognition, and adhesion [14], immunomodulation [15], and host-pathogen interaction [16]. *N*-glycans are increasingly considered potential biomarkers due to their high sensitivity to pathophysiological alterations in a diverse range of disorders, including neurological conditions such as Parkinson’s and Alzheimer’s disease [17,18]. In neurotoxic processes, deviations from normal glycosylation states can disrupt the structure and function of proteins, rendering them prone to misfolding and aggregation [19]. These aberrations can compromise cellular homeostasis and trigger cascades of pathological events, culminating in neuronal dysfunction and degeneration [19].

Humans and rats display notable differences in glycosylation, particularly concerning sialic acid modifications, notably the expression of N-glycolylneuraminic acid (NeuGc) and N-acetylneuraminic acid (NeuAc). Rats synthesize both NeuAc and NeuGc, whereas humans express NeuAc but not NeuGc [20]. These variations have significant implications for immunological responses, cellular interactions, and various other biological processes [21]. Despite these disparities, animal models, including rats, have long been indispensable in advancing our understanding of human biology and health. Rats, in particular, are a valuable resource due to their close genetic resemblance to humans and the ease with which researchers can precisely manipulate environmental factors to explore their impact on development, behavior, and overall health [21]. By leveraging these animal models, researchers can uncover insights into complex biological phenomena that may ultimately lead to advancements in medical treatments and therapies for human diseases.

Moreover, while preclinical results may not perfectly match the human population, highly conserved mechanisms in cellular regulation, such as certain patterns of *N*-glycans, suggest the suitability of murine models for studying these processes [22]. For example, glycoproteins from murine brains exhibit a similar profile of brain-specific *N*-glycans to those found in the human brain [23]. Additionally, animal models offer advantages over clinical assays, including non-ethical procedures, controlled exposure to xenobiotics, and the ability to generate a high number of samples for statistical analysis. These factors, coupled with the accelerated metabolism of small species, allowing for shorter evaluation times, underscore the utility of animal models in biomedical research [21,24].

Liquid Chromatography-Tandem Mass Spectrometry (LC-MS/MS) stands at the forefront of glycomics analysis, offering unparalleled capabilities in unraveling the intricacies of complex glycan structures [25]. This sophisticated analytical technique provides a robust platform for the in-depth profiling of glycans, offering high sensitivity and precision [26]. In the biomarker discovery phase, LC-MS/MS plays a pivotal role by identifying and quantifying subtle variations in glycan profiles within biological samples [17,25]. This capability proves invaluable in detecting nuanced changes associated with diseases or specific physiological conditions. LC-MS/MS serves as a tool for elucidating the diverse landscape of glycans and as a gateway to uncovering potential *N*-glycans associated with GBH-derived diseases. These *N*-glycans, once identified, hold the promise to serve as indicators for disease progression or response to therapeutic interventions. With continuous advancements in glycan separation techniques and mass spectrometry technology, LC-MS/MS remains a cornerstone in advancing our understanding of the dynamic interplay of glycans in health and disease.

Parallel reaction monitoring (PRM), a targeted mass spectrometry technique, has emerged as a powerful tool for validating quantitative biomolecular analysis studies, offering enhanced sensitivity, specificity, and quantification capabilities. Targeted approaches in mass spectrometry excel at analyzing low-abundance analytes by effectively filtering desired precursors from background noise [27]. In proteomics, glycomics, and glycoproteomics analysis, PRM provides several advantages, including improved signal-to-noise ratios and reduced interference from background ions [27,28,29]. In glycomics, PRM facilitates the validation of glycan structures by precisely targeting specific glycan precursors of interest. By selecting characteristic fragment ions for each glycan, PRM enhances the specificity of analysis, enabling confident identification and quantification of glycans even in complex biological samples. Moreover, PRM allows simultaneous monitoring of multiple glycan targets within a single experiment using an orbitrap mass analyzer, offering increased throughput and efficiency [27]. This capability is particularly valuable in glycomics, where the analysis of numerous glycan structures is often necessary for comprehensive biological insights.

Glyphosate-induced neurotoxicity has been reported in both rodents and humans [4,30,31]. Our recent metabolomic analysis of chronic exposure to GBH in rats revealed alterations in spatial learning memory and differential expression of essential metabolites associated with neurological disorders, indicative of GBH-induced neurotoxicity [32]. Owing to *N*-glycans’ sensitivity to the physiological environment and their relevance in neuronal health, a highly sensitive LC-MS/MS technique was utilized in profiling rat serum *N*-glycomics after chronic exposure to GBH. The results showed a potential set of *N*-glycans to differentiate the control and the GBH-exposed groups. The results also showed sex dimorphism, for which large differences in the significant *N-*glycans were observed. Additionally, blood serum offers an easy and less invasive source of biomolecules for studying mechanisms associated with GBH toxicity. Finally, the statistically significant *N*-glycans observed in the discovery phase were validated through targeted PRM-MS/MS analysis.

## 2. Materials and Methods

### 2.1. Materials and Reagents

Ammonium bicarbonate (ABC), dimethyl sulfoxide (DMSO), sodium hydroxide (NaOH) beads, and iodomethane were acquired from Sigma-Aldrich (St. Louis, MO, USA). HPLC-grade water, acetonitrile (ACN), methanol, acetic acid, and formic acid (FA) were acquired from Fisher Scientific (Fair Lawn, NJ, USA). The enzyme Peptide-N-glycosidase F (PNGase F) was obtained from New England Biolabs (Ipswich, MA, USA). Spin columns were acquired from Harvard Apparatus (Holliston, MA, USA). The GBH used was the Rival^®^ herbicide from Monsanto (St. Louis, MO, USA).

### 2.2. Animal Study

**Figure 1:**
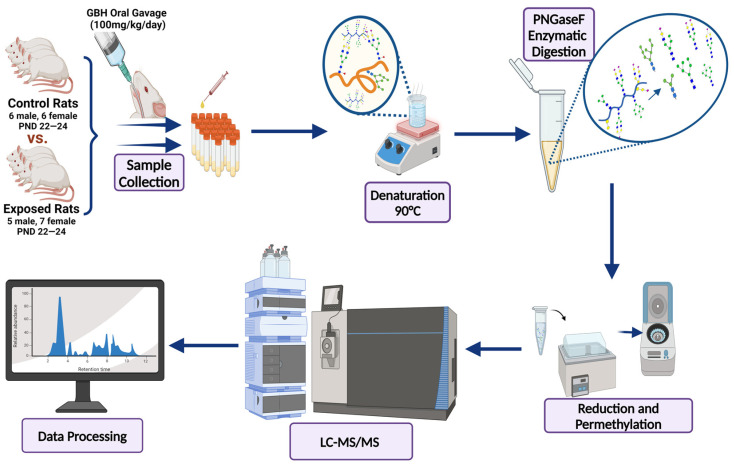
Serum *N*-glycans profiling workflow. Serum proteins were initially denatured, followed by PNGase F digestion to cleave *N*-linked glycans. The released *N*-glycans were reduced and permethylated. LC-MS/MS analysis was conducted, followed by targeted PRM analysis to validate significant *N*-glycan. Symbols: 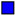, *N*-acetylglucosamine (HexNAc); 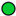, mannose (Hex); 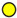, galactose (Hex); 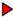, fucose; 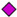, *N*-acetylneuraminic acid (NeuAc); 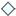, *N*-glycolylneuraminic acid (NeuGc).

A total of 24 Sprague Dawley (SD) rats, consisting of 11 males (6 control and 5 GBH-Exposed) and 13 females (6 control and 7 GBH-Exposed), were obtained from the institutional vivarium of the Autonomous University of Aguascalientes during postnatal days 22–24. The experimental techniques followed the Mexican Guidelines for Animal Care NOM-062-ZOO-1999 and the National Research Council Guide for the Care and Use of Laboratory Animals [33]. Rats were kept under controlled conditions: 12 h light/dark with light on 7:00 h, temperature of 20–22 °C, humidity of 45–55%, and food and water were available ad libitum. Twelve SD rats, five males and seven females, were allocated to the control group. The control group received daily oral gavages of injectable water at a dosage of 1 mL/kg for a duration of 12 weeks. A group of twelve SD rats, consisting of six males and six females, were designated as the GBH-treated group. They were administered GBH orally through gavages at a dosage of 100 mg of Gly/kg/day for a duration of 12 weeks. The dosage was determined based on prior findings [34,35] and was adjusted according to the rat’s weight weekly. The rats were sacrificed afterward, and their blood was collected for serum extraction. Finally, the rat serum samples were subjected to LC-MS/MS *N*-Glycomics analysis. The graphical abstract for this study is shown in Figure 1.

### 2.3. Enzymatic Release of N-Glycans

First, 10 µL of each serum sample (control and GBH-exposed) was transferred to an Eppendorf tube and diluted 10-fold with ABC buffer. The glycoproteins were subjected to denaturation in a water bath at 90 °C for 15 min. Afterward, the samples were cooled to ambient temperature, and then 1 µL of PNGase F enzyme was introduced and incubated for 18 h at 37 °C to release the *N*-glycans from the peptide backbone. After incubation, the samples were dried using a Labconco CentriVap benchtop vacuum concentrator (Kansas City, MO, USA).

### 2.4. Purification, Reduction, and Permethylation of N-Glycans

Solid SPE C18 cartridges were used for *N*-glycan purification [36,37] to eliminate unwanted matrix components, including de-glycosylated proteins and lipids. Briefly described, the dried *N*-glycan samples were reconstituted in 5% acetic acid solution. The C18 cartridges were washed with methanol and prepared with 5% acetic acid solution. The resuspended samples were applied to the C18 cartridges and washed thrice with 5% acetic acid. The flow-through from each wash was collected and dried using the vacuum concentrator. The purified *N*-glycans were reduced using 10 µL of 10 µg/µL ammonium borane complex solution and subsequent incubation at 60 °C for 1 h. Methanol was added post-incubation to eliminate residual borane by forming methyl borate, which was removed via evaporation in a vacuum concentrator. Reduced *N*-glycans were permethylated by solid-phase as described [38]. The dried, purified, and reduced *N*-glycans were reconstituted in 30 μL of DMSO, 1.2 μL of water, and 20 μL of iodomethane. NaOH beads prepared in DMSO were applied to microspin columns and centrifuged at 1.8 K rpm for 2 min. The columns were rinsed with 200 μL of DMSO and spun at 1.8 K rpm for 2 min. The prepared sample solution was applied to the microspin columns and incubated at room temperature for 25 min. Afterwards, 20 μL of iodomethane was applied to the spin columns and further incubated at room temperature for another 15 min. The permethylated *N*-glycans were released by spinning the columns at 1.8 K rpm for 2 min. The columns were subsequently rinsed with 50 μL ACN, and the total collection constituting the permethylated *N*-glycans was dried overnight and resuspended in 20% ACN and 0.1% FA for LC-MS/MS analysis.

### 2.5. LC-MS Analysis

The permethylated *N*-glycan samples were analyzed in a Dionex 3000 UltiMate nanoLC system (Thermo Scientific, Sunnyvale, CA, USA) coupled to a QExactive HF Mass Spectrometer (Thermo Scientific, San Jose, CA, USA). The *N*-glycan samples were cleaned and purified online using a C18 Acclaim PepMap trap column (75 µm × 20 mm, 3 µm, 100 Å, Thermo Scientific, Sunnyvale, CA, USA). A reversed-phase Acclaim PepMap C18 column (150 mm × 75 µm i.d.) at 55 °C was utilized for glycan separation with a 60 min gradient and 0.35 µL/min flow rate. The mobile phase A consisted of 98% HPLC water and 2% ACN, containing 0.1% FA (*v*/*v*). The mobile phase B was 100% ACN with 0.1% FA (*v*/*v*). The chromatography gradient was as follows. Initially, the mobile phase B constituted 20% of the mixture for the initial 10 min. Subsequently, there was a swift transition to 42% within a minute, followed by a gradual increase to 55% over the next 37 min. Then, there was a rapid rise to 90% over 1 min, which was sustained for 5 min, before returning to 20% over another 1 min to re-equilibrate the column for 5 min. All compounds were identified using mass spectrometry (MS) in positive ion mode. The resolution for the full MS scan was set at 60,000, covering a mass range of 480 to 1800 *m*/*z* for detecting precursor ions. The top 20 most abundant precursor ions from the full MS scan underwent High-Energy Collisional Dissociation (HCD) fragmentation with a normalized collision energy of 23 and an activation time of 50 ms.

### 2.6. LC-PRM-MS Data Validation

A targeted PRM approach was applied to validate the differentially expressed *N*-glycans between the studied groups. A transition list was compiled for PRM analysis, incorporating the *N-*glycan details observed during the discovery phase: molecule names, precursor *m*/*z* values, and retention times. Consistency was maintained by utilizing the same gradient as used in the untargeted glycomics analysis. Precursor retention times, initially derived from untargeted glycomics analysis, were meticulously cross-verified against raw data from a pooled sample using Xcalibur version 4.2 software. Theoretical fragment ions of permethylated *N*-glycans were computed utilizing Glycoworkbench 2.1. Subsequently, a transition list was compiled and utilized for the quantitative analysis of the PRM data using Skyline software version 21.2.0.536.

### 2.7. Data Analysis

The *N*-glycans and their derived adducts observed in the samples were manually checked and confirmed prior to peak integration using FreeStyle 1.4 SP2 software, and their MS/MS spectra were verified, also taking note of their retention time. This enabled *N*-glycan authentication and a true peak before quantitation using Skyline software version 21.2.0.536. The relative abundance of the identified *N*-glycans was determined manually from the absolute abundance extracted from Skyline. The relative abundance was obtained by normalizing the peak area of each identified *N*-glycan to the total abundance. These relative abundance values were used to perform an unsupervised principal component analysis (PCA) using OriginPro2022b software. The quantified *N*-glycans were analyzed statistically using the Mann-Whitney U test and subsequently subjected to the Benjamini-Hochberg Procedure to reduce the false discovery rate (FDR). *N*-glycans with a *p*-value less than 0.05 after the statistical test and correction were considered statistically significant. The heatmap was done using Genesis software version 1.8.1. Additionally, receiver operating characteristic curves (ROC) and area under curves (AUC) values were generated using SPSS^®^ version 28 (IBM) software to assess the predictive capability, selectivity, and sensitivity of each statistically significant *N*-glycan. R version 4.3.1 software was used to generate bar graphs, box plots, and Venn plots.

## 3. Results

### 3.1. N-Glycan Profiling in Control and GBH-Exposed Groups

**Figure 2:**
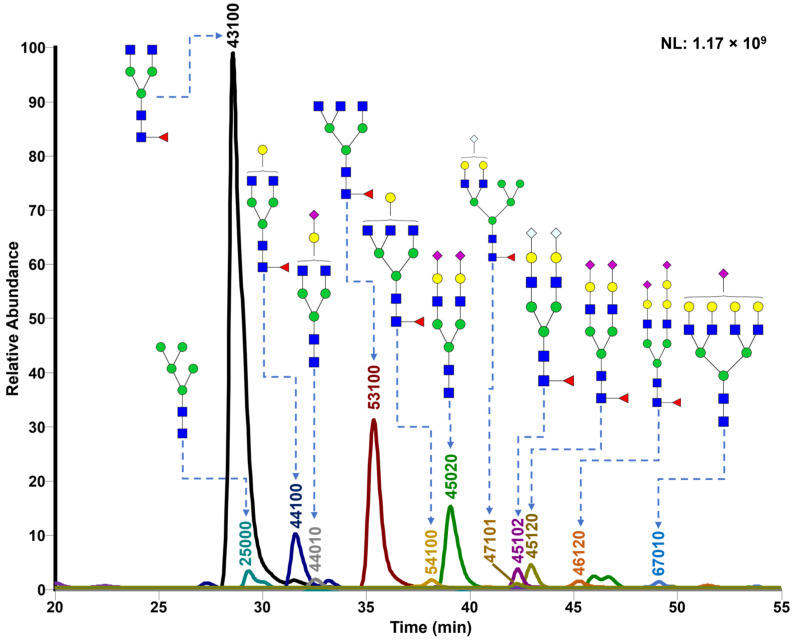
Extracted Ion Chromatogram (EIC) of representative *N*-glycans from pooled GBH samples. Representative glycan structures were assigned to each peak. *N-*Glycan nomenclature as described in Figure 1.

To investigate serum *N*-glycan alterations in rats exposed to GBH, the graphical abstract as illustrated in Figure 1 was utilized. A total of 24 rats were included in the study, comprising 12 control and 12 GBH-exposed rats. Within the experimental groups was a gender distribution of 11 males and 13 females. Blood serum was obtained from the rats, and the serum glycoproteins were subjected to denaturation; *N*-glycans were enzymatically released through PNGase F digestion. The released *N*-glycans were subjected to reduction and permethylation and were subsequently analyzed using a highly sensitive LC-MS/MS technique. The *N*-glycan profiles of the control and GBH-exposed groups were examined and compared utilizing various analytical and statistical tools. The annotation of the different *N*-glycan structures employed a five-digit nomenclature, with each digit corresponding to the number of monosaccharide units associated with the *N*-glycan structure. For example, an *N*-glycan structure is represented as “1-2-1-1-2”. The sequence of numbering included *N*-acetylglucosamine, hexose, fucose, *N*-acetylneuraminic acid, and *N*-glycolylneuraminic acid (HexNAc, Hex_2_, Fuc, NeuAc, NeuGc_2_), respectively. The *N*-glycan symbols used in this study are detailed in the caption for Figure 1. The *N*-glycan identification process is described in Figure S1 using the representative sialylated *N*-glycan, HexNAc_4_, Hex_6_, NeuAc (4-6-0-1-0) Figure S1a shows the Extracted Ion Chromatogram (EIC) of the representative *N*-glycan with the red and blue overlaid chromatogram, representing control and GBH-exposed samples, respectively. The inset provides the MS spectrum of the *N*-glycan structure, while Figure S1b shows the MS/MS glycan spectra. Overall, a total of 113 *N*-glycans were identified across the control and GBH-exposed cohorts. The peak area of the *N*-glycans was used for data normalization, and then the *N-*glycan relative abundance was calculated. Tables S1 and S2 show the relative abundance of the control and GBH-exposed samples, respectively. Figure 2 shows the sample representative extracted ion chromatogram (EIC).

### 3.2. Unsupervised PCA for Comparative N-Glycomics Analysis

**Figure 3:**
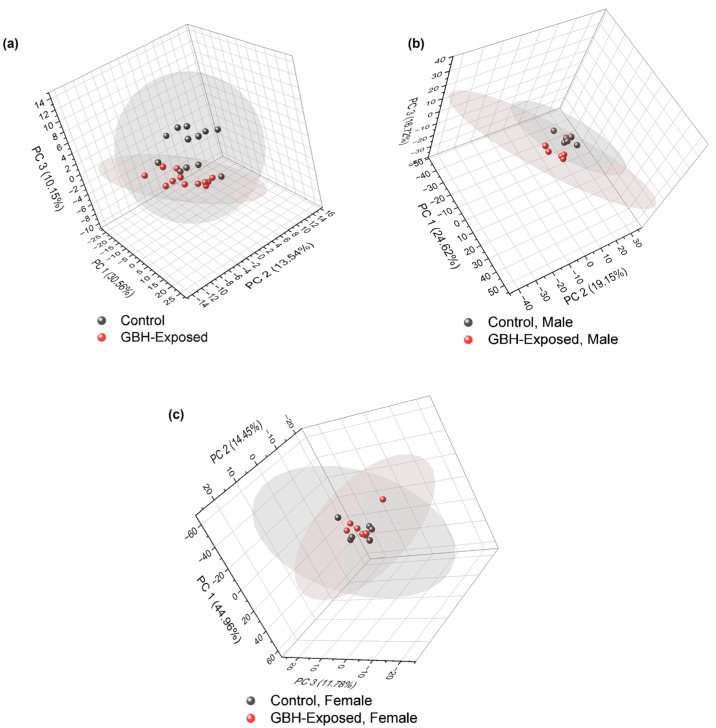
PCA of all identified *N*-glycans in control vs. GBH-exposed cohorts (**a**) total, (**b**) male only, and (**c**) female only.

The relative abundance of the 113 *N*-glycans identified in the control and GBH-exposed groups, as detailed in Tables S1 and S2, respectively, was used to perform an unsupervised Principal Component Analysis (PCA) with a 95% confidence level. The resulting PCA plots are depicted in Figure 3a–c for the total, male, and female gender subgroups. These show distinct *N*-glycan clustering patterns between the control and GBH-exposed cohorts, indicative of differential *N*-glycan expression patterns.

### 3.3. N-Glycan Heterogeneity

*N*-Glycosylation diversity across the different cohorts was investigated by sorting the *N*-glycans based on their monosaccharide composition into high mannose, sialylated NeuAc, sialylated NeuGc, fucosylated, sialofucosylated, and others. The distribution of the *N*-glycan types is shown in Figure S2 for the total, male, and female groups, respectively. The fucosylated *N*-glycan type exhibited the highest relative abundance across all three groups, followed by sialofucosylated, sialylated NeuAc, sialylated NeuGc, high mannose, and others. The Mann-Whitney U test was used to assess the differences between *N*-glycan types. The results indicated no statistically significant difference between most *N*-glycan types. However, sialylated NeuAc showed statistical significance (*p* < 0.05) specifically within the total and male groups, as shown in Figure S2a,b, respectively. These observed significant differences in the sialylated NeuAc *N-*glycan type imply a notable impact of GBH exposure on this glycan type.

### 3.4. Differentially Expressed N-Glycans

The 113 *N*-glycans identified in the samples were subjected to the Mann-Whitney U statistical test and, subsequently, the Benjamini-Hochberg (BH) Procedure. The BH procedure is pivotal in statistical analysis because it reduces the false discovery rate and prevents the erroneous rejection of a true null hypothesis caused by chance variations in *p*-values. After the statistical procedures, 18 *N-*glycans were statistically significant between the control relative to the GBH-exposed cohort in the total, 11 in the male comparison, and 1 in the female comparison. Table 1 and Table 2 show the significant *N*-glycans within the total, male, and female groups, respectively, including details such as the *N*-glycan composition, putative *N*-glycan structure, *p*-values, adjusted *p*-value based on the BH procedure, and Fold Change (FC). In the total group, 9 *N*-glycans increased in expression, while 9 decreased in expression. Within the male gender subgroup, 5 *N*-glycans increased in expression, and 6 decreased in expression. However, in the female gender subgroup, only one *N*-glycan was found to have a significant increase in expression. Detailed information is provided in Table 1 and Table 2.

### 3.5. PRM Validation

The statistically significant *N*-glycans (*p* < 0.05) in the full scan were validated using targeted PRM analysis, and they all showed the same expression pattern as the full scan data. The details, including the targeted precursor m/z values, transition ion fragments, Full Scan Fold Change (FC), Log2FC and PRM FC, and Log2FC, are shown in Tables S3 and S4 for the total, male, and female gender subgroups, respectively. The PRM analysis was utilized to validate the expression of the significant *N*-glycans in the control vs. GBH-exposed cohorts. This further affirms the reliability of these *N*-glycans in distinguishing GBH exposure.

### 3.6. Expression Analysis/Cluster Heatmaps

**Figure 4:**
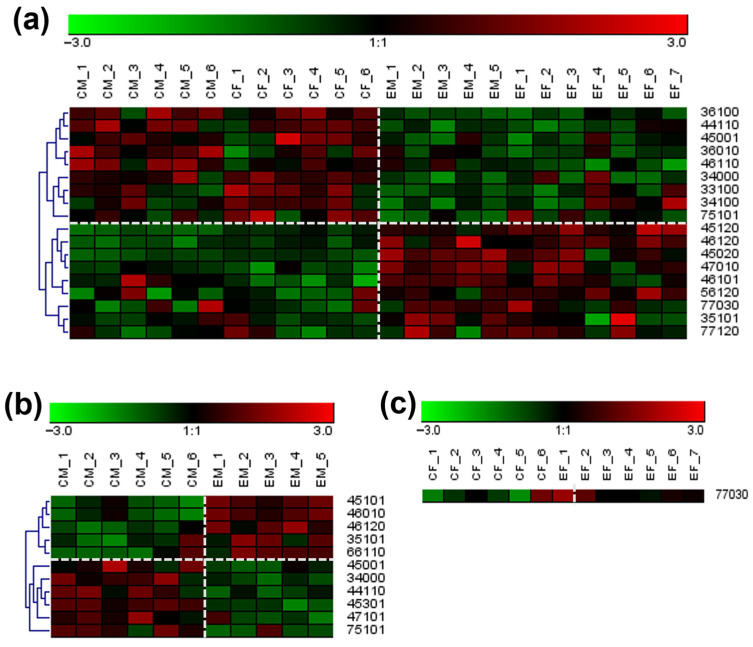
Heatmap of statistically significant and PRM validated *N*-glycans in control vs. GBH-exposed cohort in (**a**) total, (**b**) male, and (**c**) female gender subgroups. The red and green colors depict the increase or decrease in expression of the *N*-glycans, respectively. CM, EM, CF, and EF represent Control_Male, Exposed_Male, Control_Female, and Exposed_Female, respectively. *N-*Glycan nomenclature and symbols as described in Figure 1.

The significant PRM-validated N-glycans within the control and GBH-exposed cohorts were investigated using heat maps to analyze their expression patterns. Figure 4a–c depicts the heatmaps, illustrating *N*-glycan expression in the total, male, and female groups, respectively. These heatmaps elucidate spatial relationships and correlations between the control and GBH-exposed cohorts, utilizing a color gradient spanning from bright red to green. The bright red and green hues are visual indicators of the *N-*glycan increase or decrease in its expression, respectively. The rows in the heatmaps correspond specifically to statistically significant and PRM-validated *N*-glycans, while the columns meticulously represent biological replicates within both the control and GBH-exposed cohorts. The heatmaps show distinct trends and clusters in *N*-glycan expression within the cohorts. This observation affirms changes in molecular interactions and regulatory networks of *N*-glycan expression due to GBH exposure in the control and GBH-exposed cohorts.

### 3.7. Box Plots and ROC Analysis

**Figure 5:**
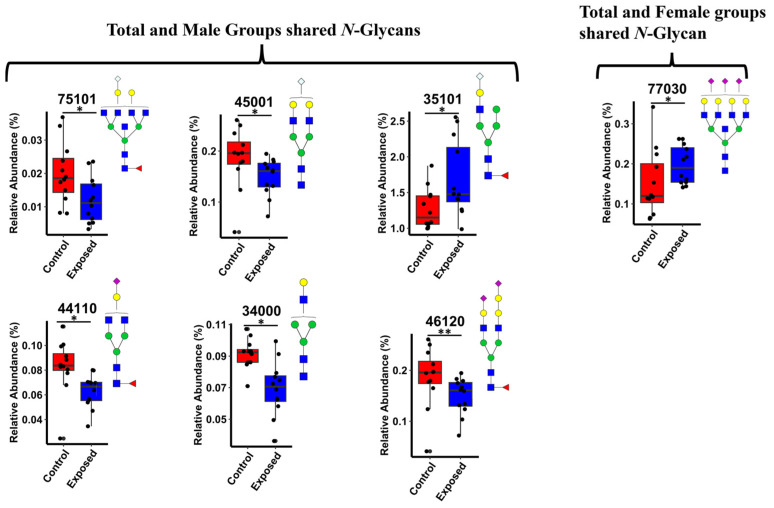
Box plots of the significant *N-*Glycans shared across the total, male, and female groups. Statistical significance was performed using the Mann–Whitney U test followed by a BH correction procedure (* < 0.05, ** < 0.01). *N-*Glycan nomenclature as described in Figure 1.

Figure S3 illustrates the shared and distinct significant *N*-glycans among the total, male, and female groups through a Venn plot. Within the total group, 11 *N*-glycans (47.8%) are exclusive, while the male group exhibits 5 unique *N*-glycans (21.7%). Additionally, 6 *N*-glycans (26.1%) are present in the total and male groups, and 1 *N*-glycan (4.3%) is shared between the total and female groups. The shared *N*-glycans in total and male or female are HexNAc_7_,Hex_5_,Fuc,NeuGc (7-5-1-0-1), HexNAc_4_,Hex_5_,NeuGc (4-5-0-0-1), HexNAc_3_,Hex_5_,Fuc,NeuGc (3-5-1-0-1), HexNAc_4_,Hex_4_,Fuc,NeuAc_,_ (4-4-1-1-0), HexNAc_3_,Hex_4_, (3-4-0-0-0), HexNAc_4_,Hex_6_,Fuc,NeuAc_2_ (4-6-1-2-0), and HexNAc_7_,Hex_7_, NeuAc_3_, (7-7-0-3-0). These are presented using box graphs in Figure 5. The box graphs for the unique *N*-glycans in the total and male gender subgroups are shown in Figure S4.

**Figure 6:**
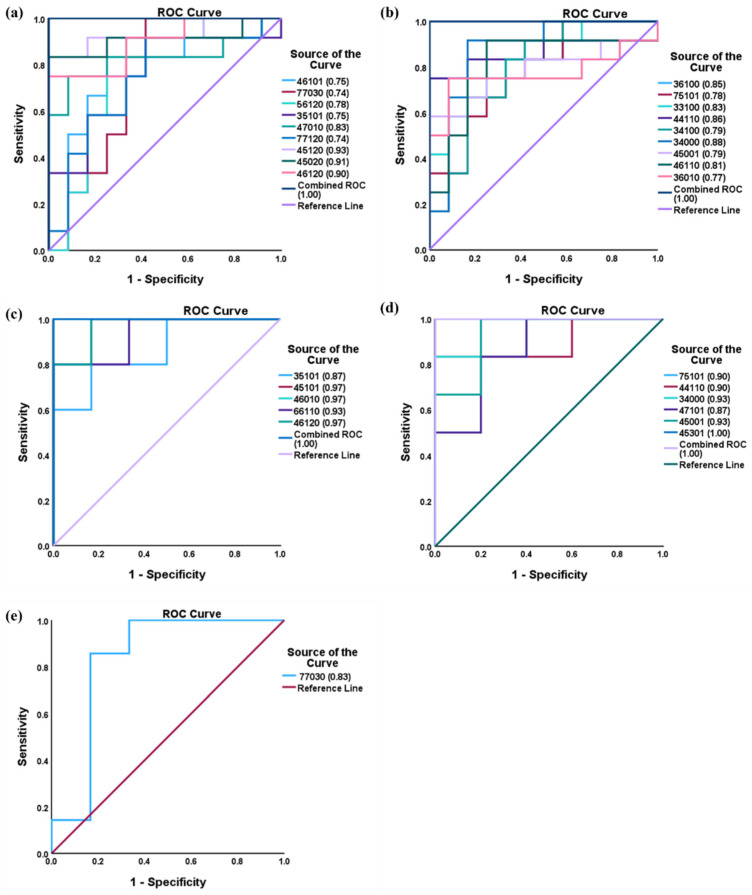
ROC/AUC curves of the significant *N-*Glycans in the (**a**) Total group ROC curve for *N-*glycans with increased expression, (**b**) Total group ROC curve for *N-*glycans with decreased expression, (**c**) Male group ROC curve for *N-*glycans with increased expression, (**d**) Male group ROC curve for *N-*glycans with decreased expression, and (**e**) Female group ROC curve for the differentially *N-*glycan, having increased expression. *N-*Glycan nomenclature as described in Figure 1.

A receiver operating characteristic (ROC) curve was utilized, as illustrated in Figure 6. The ROC curve distinguishes between the control and GBH-exposed cohorts by plotting sensitivity against specificity. Figure 6a,b show the ROC curve for the total *N-*glycan groups that increase and decrease in expression, with AUC values ranging from 0.74 to 0.93 and 0.77 to 0.88, respectively. Similarly, Figure 6c,d show the male group’s significant number of *N*-glycans increasing in expression with AUC values ranging from 0.87 to 0.97, and the *N-*glycans decreasing in expression with AUC values between 0.87 and 1.00. Additionally, the female group’s significant *N*-glycan has an AUC value of 0.83, Figure 6e. The observed AUC values were above 0.7 for all the statistically significantly expressed *N*-glycans in all three groups, suggesting their potential to distinguish differences between the studied groups. Notably, a combined AUC value of 1.0 was found across all groups, indicating a robust discriminatory ability of the reported *N*-glycans when considered collectively.

## 4. Discussion

The widely used herbicide glyphosate, which inhibits the shikimate pathway involved in the synthesis of aromatic amino acids in plants, was previously considered harmless to humans due to the absence of this pathway [3,30]. Recent studies have drawn attention to the potential neurotoxic effects of GBH in both rodents and humans [4]. GBH exposure has been linked to changes in neurobehavioral patterns, likely stemming from disruptions in neuronal development [39]. Our previous investigation revealed that prolonged exposure to GBH disrupts the expression of metabolites such as epinephrine, L-arginine, and D-arginine, which play key roles in neuroregulation [32]. This disruption consequently impacts spatial learning memory in young rats [32]. Additionally, our findings indicate that GBH inhibits the histamine degradation pathway while activating the citrulline biosynthesis pathway. These pathways are associated with neuroinflammation and immune responses, which may be potential outcomes of glyphosate toxicity [32].

However, *N*-linked glycans, a highly heterogeneous and tightly regulated form of glycosylation, exhibit diverse structures and functions within mammalian cells [40]. The synthesis of *N*-glycans is governed by a complex process involving various glycosyltransferases and glycosidases [41]. In this study, we utilized *N*-glycomics profiling techniques to identify *N*-glycan changes associated with GBH-induced toxicity using minimally invasive biofluids such as blood serum. Our findings hold the potential for translation into human studies to address changes associated with GBH exposure.

The investigation of *N*-glycome changes was conducted in both male and female rats with the aim of providing a comprehensive understanding of these changes across genders. An unsupervised PCA was initially used to examine the clustering pattern in the data. Unsupervised PCA analysis is helpful in omics research for global analysis of datasets, providing entirely unsupervised information on the main directions of highest variability in the data, allowing comparisons between samples or clustering [42]. The PCA results, as shown in Figure 3, suggest differences between the glycomes of control and GBH-exposed in the total group and the gender subgroups, with the total and male gender subgroups showing more separation between the control and GBH-exposed. Notably, the total and male gender subgroups exhibited more distinct separation between the control and GBH-exposed groups, indicating significant differences in *N*-glycan profiles.

The 113 identified *N*-glycans were classified according to their monosaccharide composition, resulting in distinct categories of high mannose, sialylated NeuAc, sialylated NeuGc, fucosylated, sialofucosylated, and others. Notably, the sialylated NeuAc *N*-glycan type exhibited a significant difference, specifically within the total and male gender subgroups with *p*-values less than 0.05, Figure S2. This finding indicates a major impact of GBH exposure on sialylated NeuAc *N*-glycans. Sialylated *N*-glycans have one or more sialic acid monosaccharides in their structure. Sialic acids are negatively charged at physiological pH, consisting of over 30 derivatives, with NeuAc and NeuGc being the major derivatives [43]. Sialic acids can undergo modifications such as acetylation, sulfation, and others, influencing a wide range of protein-protein interactions, conformation, oligomerization, and interactions with the extracellular matrix [44]. Owing to the dynamic nature of sialic acids, they are highly prone to expression changes during development and in diseases like immune disorders and cellular toxicity [45]. Moreover, sialylated *N*-glycans serve as binding partners for lectins such as selectins and siglecs, which regulate important physiological and pathological processes [46]. The significant difference observed in sialylated NeuAc *N*-glycans between the control and GBH-exposed groups, as depicted in Figure S2, suggests a pronounced impact of GBH exposure on NeuAc sialylated *N*-glycans. Of note, NeuAc sialic acid is expressed in most mammals, including humans [47], hinting at the potential repercussions of GBH toxicity on NeuAc in humans post-exposure to GBH.

Statistical analysis of 113 *N*-glycans identified in the 24 rat serum samples revealed significant changes in 18 *N*-glycans following the Benjamini-Hochberg correction. Of these, 9 showed an increase in expression, while 9 exhibited decreased expression, as shown in Table 1. In the male and female gender groups, 11 and 1 *N*-glycans, respectively, were identified as significant after correction, as shown in Table 2. Heatmaps were employed to visually compare the serum abundances of *N*-glycans between the control and GBH-exposed rat groups. Figure 4a illustrates the heatmap for the total group, while Figure 4b,c show the heatmaps for the male and female groups, respectively. Notably, most of the differentially expressed *N*-glycans across the groups were sialylated and sialofucosylated *N*-glycans, suggesting a more pronounced impact of exposure on these *N*-glycan types. These results indicate a greater number of *N*-glycan changes in male rats compared to female rats following chronic exposure to GBH. This implies that females exhibit lower susceptibility to *N*-glycan alterations induced by GBH toxicity compared to males. In our previous investigation on the serum metabolome of rats exposed to GBH [32], we observed more significant changes in female than male rats. In this study, we have identified substantial differences in the serum glycome of male rats. Metabolites are functional products of interactions between the host and various factors such as environment, disease state, or clinical information, while proteins are direct products of the genome [48]. The results from the previous metabolome study [32] and this glycome investigation strongly suggest significant gender differences in the toxic effects of GBH in rats. Other investigations have also reported the profound impact of sex dimorphism on the expression of glycans and metabolites [49,50,51,52].

Human congenital glycosylation disorders have consistently shown that deficits in *N*-glycosylation, specifically sialylated *N*-glycans, lead almost invariably to significant neurological impairments, underscoring the critical role of this post-translational modification (PTM) in the nervous system [11]. Among the total group, 14 out of 18 differentially expressed *N*-glycans (*p*-value < 0.05) contain sialic acid in their structures, either NeuAc or NeuGc. In the male gender subgroup, 10 out of 11 differentially expressed *N*-glycans contain sialic acid, while the only differentially expressed *N*-glycan in the female group is a sialylated *N*-glycan (Table 1 and Table 2). These findings suggest a potential network of GBH toxicity in this type of *N*-glycan. Neuronal signaling is a complex molecular process crucial for memory consolidation, social behavior, and decision-making [53]. *N*-glycans, observed on numerous proteins, are vital to modulating neuronal signaling. While not all *N*-linked glycan modifications are essential, sialylated *N*-glycans have a profound impact on neuronal activity [53]. Sialylated *N*-glycans on voltage-gated ion channels (VGICs) are identified as potent modulators, significantly influencing axon firing rates [54]. The changes in sialylated *N*-glycans observed between the control and GBH-exposed groups, as indicated by the current findings, could potentially impact the network of neuronal signaling. This could also be related to the observed loss of spatial learning memory in GBH-exposed rats, as reported in our previous analysis [32]. Also, numerous proton pumps and neurotransmitter antiporters situated within synaptic vesicles are characterized by the presence of sialylated glycoproteins [55]. The differential expression of sialylated *N*-glycans in the control relative to the GBH-exposed group suggests a plausible pathway through which chronic exposure to GBH may induce neurotoxic effects, impacting neuronal signaling dynamics.

Another mechanism by which glyphosate exerts neurotoxic effects is through neuroinflammation and oxidative stress [4]. Glyphosate exposure triggers the rapid onset of oxidative stress, characterized by heightened lipid peroxidation (LPO) attributed to free radical activity [56]. Inflammation is a physiological process that aids the immune system in managing specific pathological conditions. However, prolonged inflammation could harm the CNS, causing neuronal death [57]. Variations in the patterns of sialylation and fucosylation have been documented in conditions affecting the central nervous system and during instances of neuroinflammation [58,59]. Our findings reveal significant changes in multiple sialylated and fucosylated *N*-glycans, as shown in Table 1 and Table 2, possibly linked to neurotoxic effects resulting from glyphosate exposure. It is noteworthy that brain-targeted inactivation of β-1,6-N-acetylglucosaminyltransferase (GlcNAcT)-I, which is necessary to produce hybrid and complex *N*-linked glycans, causes severe neurological symptoms such as locomotor abnormalities, tremors, and paralysis [19,60,61]. Consistent with these findings, our results demonstrate alterations in hybrid and complex type *N*-glycans following chronic exposure to GBH. Specifically, we identified a decrease in the expression of various complex type *N*-glycans, including HexNAc7,Hex5,Fuc,NeuGc (7-5-1-0-1);HexNAc4,Hex5,NeuGc (4-5-0-0-1); HexNAc4,Hex4,Fuc,NeuAc (4-4-1-1-0); HexNAc4,Hex6,Fuc,NeuAc2 (4-6-1-2-0); and hybrid *N*-glycan HexNAc3,Hex6,NeuAc (3-6-0-1).

After the nontargeted analysis, the significant *N*-glycans (*p* < 0.05) were validated using targeted PRM analysis. PRM is a targeted mass spectrometry technique that utilizes the high-resolution capability of the Orbitrap mass analyzer to detect all product ions from a preset precursor ion. It overcomes the need to preselect fragment ions in multiple reaction monitoring (MRM) and enables the detection of all fragment ions, thereby allowing accurate quantification of targeted analytes [27]. PRM is a valuable tool that facilitates the validation of analytical measurements of the proposed *N*-changes associated with GBH exposure [27]. The full scan and PRM analysis have the same expression pattern for the differentially expressed *N*-glycans.

The Venn plot depicted in Figure S3 visually represents the shared and distinct significant *N*-glycans across the total, male, and female gender subgroups. This graphical illustration provides valuable insights into the overlap and uniqueness of identified *N*-glycans within and between these distinct cohorts. Through intersecting circles, the plot is aimed at delineating the specific *N*-glycans that are common to multiple groups as well as those that are exclusive to individual groups. This analysis facilitates the understanding of the glycan profiles within each subgroup and highlights potential similarities and differences in glycan expression patterns across genders. Figure 5 shows box plots of the shared *N*-glycans between the total and male gender subgroups, including HexNAc_7_,Hex_5_,Fuc,NeuGc (7-5-1-0-1), HexNAc_4_,Hex_5_,NeuGc (4-5-0-0-1), HexNAc_3_,Hex_5_,Fuc,NeuGc (3-5-1-0-1), HexNAc_4_,Hex_4_,Fuc,NeuAc (4-4-1-1-0), HexNAc_4_,Hex_6_,Fuc,NeuAc_2_ (4-6-1-2-0). The shared *N*-glycan between the total and female gender subgroups is HexNAc_7_,Hex_7_,NeuAc_3_, (7-7-0-3-0), also shown in Figure 5. Overall, sialylated and sialofucosylated *N*-glycans represent the predominant types among the significant *N*-glycans. These specific glycan types have been identified as essential drivers of neuronal health [53], underscoring their involvement in neurotoxicity associated with glyphosate exposure, as indicated by these research findings.

Upon validating the expression of significant *N*-glycans using PRM analysis, we utilized ROC curves to assess the *N*-glycans potential ability to distinguish changes associated with GBH-exposure. The ROC curve methodology facilitates a thorough evaluation of the reliability of these *N*-glycans in terms of sensitivity and specificity across both control and GBH-exposed groups. In the total group, the significant *N*-glycans exhibited AUC values ranging from 0.74 to 0.93, indicating promising discriminatory ability, as shown in Figure 6a,b. Similarly, the male and female gender subgroups displayed AUC values of 0.87 to 0.97 (Figure 6c,d) and 0.83 (Figure 6e), respectively. The total and male gender subgroups have a combined AUC of 1 for both up- and down-regulated *N*-glycans. This collective efficacy suggests that, when analyzed as a unified set, these *N*-glycans possess the capability to effectively distinguish between control and GBH-exposed cohorts across various thresholds. This finding captures the discriminatory power of these *N*-glycans, further supporting their potential utility in tracking serum changes associated with GBH exposure.

Given the crucial role of glycans in maintaining cellular homeostasis [12,13,14,15], investigating changes in glycome profiling resulting from exposure to potentially harmful agents is essential. In this regard, toxico-glycomics emerges as a promising avenue within the glycomics field, offering insights into novel biochemical pathways affected by intoxications. While some studies have examined the impact of xenobiotic exposure on glycome profiling [62,63], there remains a significant gap in understanding the potential neurological implications of herbicide exposure through toxico-glycomics analysis.

## 5. Conclusions

This research marks the first comprehensive exploration of the impact of GBH exposure on the *N*-glycan composition in rat blood serum. A highly sensitive LC-MS/MS glycomics profiling technique was employed in this analysis. The results from this investigation reveal that GBH exposure induces notable alterations in the *N*-glycan profile of blood serum. Interestingly, male rats exhibited a higher susceptibility to these changes compared to their female counterparts. A significant portion of the differentially expressed *N*-glycans falls within the categories of sialylated and sialofucosylated *N*-glycans including HexNAc_7_,Hex_5_,Fuc,NeuGc (7-5-1-0-1), HexNAc_4_,Hex_5_,NeuGc (4-5-0-0-1), HexNAc_3_,Hex_5_,Fuc,NeuGc (3-5-1-0-1), HexNAc_4_,Hex_4_,Fuc,NeuAc (4-4-1-1-0), HexNAc_4_,Hex_6_,Fuc,NeuAc_2_ (4-6-1-2-0), and HexNAc_7_,Hex_7_,NeuAc_3_, (7-7-0-3-0). Sialylated and sialofucosylated *N*-glycans have been implicated in various neurotoxic and neurological conditions. The observed changes in these *N*-glycan types suggest a potential neurotoxic effect resulting from chronic GBH exposure. The identification of these altered *N*-glycans provides insight that can contribute to a deeper understanding of the mechanisms underlying GBH-induced toxicity and its potential implications for neurological health.

## Figures and Tables

**Table 1:** Statistically significant *N-*glycans between control relative to GBH-Exposed cohort in the Total group with their percentage relative abundance in the control and GBH-Exposed (average ± standard deviation), *p*-value, adjusted *p*-value, and Fold Change (FC). Statistical analysis was performed using the Mann-Whitney U test. To control the false discovery rate, the Benjamini-Hochberg correction was applied to obtain the adjusted *p*-values. A total of 18 *N*-glycans were significant. Of these, nine showed an increase in expression, while nine exhibited decreased expression. *N-*glycan nomenclature and symbols as described in Figure 1.

*N*-Glycan Composition	Putative *N*-GLYCAN Structure	Control	GBH- Exposed	*p*-Value	Adjusted *p*-Value *	Fold Change (FC)
4-5-1-2-0	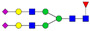	0.7 ± 0.2	1.1 ± 0.3	0.0003	0.005	1.59
4-5-0-2-0	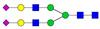	2.4 ± 0.9	4 ± 1	0.0007	0.006	1.68
4-6-1-2-0	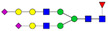	0.16 ± 0.06	0.27 ± 0.06	0.001	0.007	1.72
4-7-0-1-0	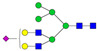	0.07 ± 0.03	0.11 ± 0.04	0.007	0.01	1.36
5-6-1-2-0	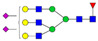	0.04 ± 0.01	0.06 ± 0.01	0.02	0.03	1.32
3-5-1-0-1	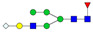	1.2 ± 0.2	1.5 ± 0.5	0.04	0.04	1.33
4-6-1-0-1	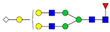	0.03 ± 0.01	0.05 ± 0.02	0.04	0.04	1.24
7-7-0-3-0	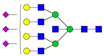	0.13 ± 0.06	0.17 ± 0.02	0.04	0.04	1.30
7-7-1-2-0	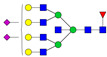	0.04 ± 0.02	0.06 ± 0.01	0.04	0.04	1.46
3-4-0-0-0	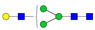	0.091 ± 0.009	0.07 ± 0.02	0.002	0.01	0.76
4-4-1-1-0	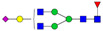	0.08 ± 0.03	0.06 ± 0.02	0.002	0.01	0.75
3-6-1-0-0	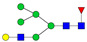	0.02 ± 0.01	0.010 ± 0.001	0.003	0.01	0.59
3-3-1-0-0	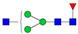	1.0 ± 0.2	0.7 ± 0.2	0.006	0.01	0.75
3-4-1-0-0	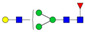	0.09 ± 0.02	0.07 ± 0.02	0.01	0.02	0.75
4-5-0-0-1	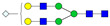	0.17 ± 0.08	0.15 ± 0.04	0.01	0.02	0.80
4-6-1-1-0	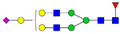	5 ± 1	4.3 ± 0.4	0.01	0.01	0.84
3-6-0-1-0	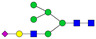	0.6 ± 0.2	0.61 ± 0.09	0.02	0.03	0.91
7-5-1-0-1	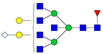	0.020 ± 0.009	0.012 ± 0.007	0.02	0.03	0.60

* Based on Benjamini-Hochberg (BH) Procedures.

**Table 2:** Statistically significant *N*-glycans in control relative to GBH-Exposed cohort in male and female gender subgroups with their percentage relative abundance in the control and GBH-Exposed (average ± standard deviation), *p*-value, adjusted *p*-value, and Fold Change (FC). In the male and female gender groups, 11 and 1 *N*-glycans, respectively, were significant. Statistical analysis was performed using the Mann-Whitney U test. To control the false discovery rate, the Benjamini-Hochberg correction was applied to obtain the adjusted *p*-values. *N*-Glycan nomenclature and symbols as described in Figure 1.

*N*-Glycan Composition	Putative *N*-Glycan Structure	Control	GBH- Exposed	*p*-Value	Adjusted *p*-Value *	Fold Change (FC)
3-5-1-0-1	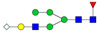	1.4 ± 0.3	2.0 ± 0.5	0.04	0.04	1.45
4-5-1-0-1	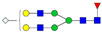	0.06 ± 0.02	0.10 ± 0.01	0.01	0.03	1.65
4-6-0-1-0	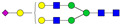	0.06 ± 0.02	0.10 ± 0.01	0.01	0.03	1.67
4-6-1-2-0	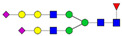	0.17 ± 0.03	0.30 ± 0.09	0.01	0.02	1.80
6-6-1-1-0	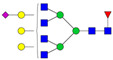	0.016 ± 0.006	0.029 ± 0.007	0.01	0.04	1.76
3-4-0-0-0	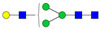	0.090 ± 0.009	0.07 ± 0.01	0.01	0.04	0.73
4-5-0-0-1	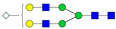	0.20 ± 0.03	0.15 ± 0.03	0.01	0.03	0.77
4-4-1-1-0	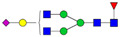	0.09 ± 0.02	0.06 ± 0.01	0.02	0.04	0.71
7-5-1-0-1	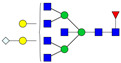	0.016 ± 0.006	0.008 ± 0.006	0.02	0.03	0.49
4-5-3-0-1	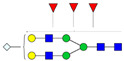	3.4 ± 0.2	2.7 ± 0.2	0.006	0.03	0.78
4-7-1-0-1	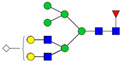	0.24 ± 0.04	0.18 ± 0.04	0.04	0.04	0.74
7-7-0-3-0 **	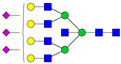	0.13 ± 0.06	0.18 ± 0.04	0.04	0.04	1.41

* Based on Benjamini-Hochberg (BH) Procedure ** Female gender subgroup.

## Data Availability

The mass spectrometry data has been uploaded to GlycoPost [64] with accession number GPST000403.

## Supplementary Materials

The following supporting information can be downloaded at: https://www.mdpi.com/article/10.3390/biom14091077/s1, Table S1: Relative abundances of *N*-glycans derived from the Control sample cohort. N-Glycan nomenclature as was described in the main manuscript. CM refers to Control Male, while CF refers to Control Female; Table S2: Relative abundances of N-glycans derived from GBH-Exposed sample cohort. *N*-Glycan nomenclature as was described in the main manuscript. EM refers to Exposed Male, while EF refers to Exposed Female; Table S3: N-Glycans in the total rat group validated by LC-PRM-MS, including precursor m/z, transition fragment ions, fold change (FC) and log2FC for the full scan and PRM validation. N-Glycan nomenclature as was described in the main manuscript; Table S4: *N*-Glycans in the rat gender subgroup validated by LC-PRM-MS, including precursor m/z, transition fragment ions, fold change (FC), and log2FC for the full scan and PRM validation. N-Glycan nomenclature as was described in the main manuscript; Figure S1: N-glycan identification process: (a) Extracted Ion Chromatogram (EIC) of sialylated N-glycan, HexNAc4,Hex6,NeuAc (4-6-0-1-0) with the red and blue overlayed chromatogram extracted from control and GBH-exposed samples respectively. The inset provides the full MS spectrum of the N-glycan structure. (b) The MS/MS spectrum of the 4-6-0-1-0 with fragment ions labeled next to their corresponding peaks. *N*-Glycan symbols and nomenclature as described in Figure 1; Figure S2. Distribution of all identified *N*-glycan by types between control vs. GBH-exposed cohorts in (a) total, (b) male, and (c) female gender subgroups. Statistical significance was performed using Mann–Whitney U test (* < 0.05); Figure S3. Venn plot shows unique and overlapping significant N-glycans in the total and gender subgroup data sets of comparison between control and GBH-exposed rats. N-Glycan nomenclature as described in Figure 1; Figure S4: Box plots of the unique significant N-glycans: (a) Unique N-glycans in the total group, and (b) unique N-glycans in the male group. Statistical significance was performed using Mann–Whitney U test followed by BH correction procedure (* < 0.05, ** < 0.01). *N*-Glycan symbols and nomenclature as described in Figure 1.
