# Supplementary material for: Serum N-Glycan Changes in Rats Chronically Exposed to Glyphosate-Based Herbicides
Source: Biomolecules. 2024 Aug 28;14(9):1077. doi: 10.3390/biom14091077 (PMC11430009; doi:10.3390/biom14091077)

Supplementary Information

Table of Contents:

Supplementary Tables:

Table S1. Relative abundances of *N*-glycans derived from the Control sample cohort. *N*-Glycan nomenclature as was described in the main manuscript. CM refers to Control Male, while CF refers to Control Female.

Table S2. Relative abundances of *N*-glycans derived from GBH-Exposed sample cohort. *N*-Glycan nomenclature as was described in the main manuscript. EM refers to Exposed Male, while EF refers to Exposed Female.

Table S3: *N*-Glycans in the total rat group validated by LC-PRM-MS, including precursor m/z, transition fragment ions, fold change (FC) and log2FC for the full scan and PRM validation. *N*-Glycan nomenclature as was described in the main manuscript.

Table S4: *N*-Glycans in the rat gender subgroup validated by LC-PRM-MS, including precursor m/z, transition fragment ions, fold change (FC), and log2FC for the full scan and PRM validation. *N*-Glycan nomenclature as was described in the main manuscript.

Supplementary Figures:

Figure S1: *N*-glycan identification process: (**a**) Extracted Ion Chromatogram (EIC) of sialylated *N*-glycan, $HexNAc_4$, Hex_6, NeuAc (4-6-0-1-0) with the red and blue overlayed chromatogram extracted from control and GBH-exposed samples respectively. The inset provides the full MS spectrum of the *N*-glycan structure. (**b**) The MS/MS spectrum of the 4-6-0-1-0 with fragment ions labeled next to their corresponding peaks. *N*-Glycan symbols and nomenclature as described in **Figure 1**.

Figure S2. Distribution of all identified *N*-glycan by types between control *vs.* GBH-exposed cohorts in (**a**) total, (**b**) male, and (**c**) female gender subgroups. Statistical significance was performed using Mann–Whitney U test (* < 0.05)

Figure S3. Venn plot shows unique and overlapping significant *N*-glycans in the total and gender subgroup data sets of comparison between control and GBH-exposed rats. *N*-Glycan nomenclature as described in **Figure 1**.

Figure S4: Box plots of the unique significant *N*-glycans: (**a**) Unique *N*-glycans in the total group, and (**b**) unique *N*-glycans in the male group. Statistical significance was performed using Mann–Whitney U test followed by BH correction procedure (* < 0.05, ** < 0.01). *N*-Glycan symbols and nomenclature as described in **Figure 1**.

Table S1. Percentage relative abundances of *N*-glycans derived from the Control sample cohort. *N*-Glycan nomenclature as was described in the main manuscript. CM refers to Control Male, while CF refers to Control Female.

N-Glycan	CM_1	CM_2	CM_3	CM_4	CM_5	CM_6	CF_1	CF_2	CF_3	CF_4	CF_5	CF_6
4-5-1-1-0	0.0169	0.0244	0.0284	0.0218	0.0234	0.0232	0.0373	0.0433	0.0291	0.0324	0.0324	0.0253
4-5-1-2-0	0.6420	0.5670	0.6880	0.6370	0.6610	0.6070	0.3010	0.5790	0.8010	0.7930	0.6560	0.7780
4-5-0-2-0	2.7500	2.3300	2.6000	2.9500	2.9100	2.8700	0.7280	2.2400	3.1900	2.9800	2.5200	2.6300
4-6-1-2-0	0.1780	0.1210	0.1700	0.1760	0.1510	0.2040	0.0523	0.1210	0.2190	0.2020	0.1320	0.2040
3-4-0-0-0	0.0930	0.0913	0.0931	0.0918	0.0972	0.0710	0.0861	0.1070	0.0932	0.0847	0.1030	0.0860
4-4-1-1-0	0.0914	0.1150	0.0831	0.1010	0.0843	0.0679	0.0246	0.0776	0.0883	0.0828	0.0992	0.0805
6-6-1-1-0	0.0149	0.0127	0.0163	0.0091	0.0204	0.0252	0.1140	0.0221	0.0200	0.0268	0.0159	0.0314
3-6-1-0-0	0.0202	0.0243	0.0107	0.0284	0.0174	0.0249	0.0020	0.0146	0.0225	0.0208	0.0178	0.0186
4-5-1-0-1	0.0552	0.0753	0.0916	0.0513	0.0509	0.0441	0.0359	0.0756	0.0892	0.0671	0.0753	0.0595
4-6-0-1-0	0.0552	0.0748	0.0913	0.0513	0.0474	0.0441	0.0359	0.0752	0.0941	0.0644	0.0778	0.0591
3-3-1-0-0	0.9260	0.9120	1.1800	0.7530	0.7270	0.6270	1.3200	1.0200	1.0400	0.8380	1.1600	0.7180
4-5-3-0-1	3.3600	3.7900	3.3200	3.4300	3.3700	3.1400	1.7300	2.9500	3.1100	3.4400	3.2600	3.2500
4-7-0-1-0	0.0969	0.0872	0.1160	0.1010	0.0937	0.0945	0.0261	0.0444	0.1070	0.0844	0.0874	0.0773
4-6-1-1-0	7.2000	7.3400	5.1200	7.3000	6.2600	6.0600	1.5000	4.9800	5.2500	5.7000	5.2800	5.3500
6-6-0-1-0	0.0343	0.0299	0.0471	0.0184	0.0534	0.0461	0.0716	0.0449	0.0353	0.0498	0.0366	0.0511
3-4-1-0-0	0.0646	0.0829	0.1210	0.0559	0.0631	0.0821	0.0998	0.0834	0.0894	0.0795	0.1060	0.0620
3-5-0-1-0	1.1000	1.0800	0.9430	1.1100	0.9520	1.2800	0.4260	1.0300	1.1000	1.1200	1.1300	1.1000
4-5-0-0-1	0.1800	0.2120	0.2500	0.1950	0.1650	0.1970	0.0411	0.1240	0.2610	0.1970	0.2350	0.1770
5-6-1-2-0	0.0329	0.0549	0.0936	0.0212	0.0477	0.0341	0.0247	0.0358	0.0317	0.0347	0.0297	0.0624
7-5-1-0-1	0.0174	0.0238	0.0182	0.0080	0.0190	0.0124	0.0267	0.0368	0.0082	0.0149	0.0342	0.0209
3-6-0-1-0	0.8290	0.7660	0.7160	0.7320	0.6860	0.8670	0.1820	0.5210	0.6940	0.6890	0.5970	0.7070
3-5-1-0-1	1.4700	0.9990	1.0200	1.4500	1.3400	1.8800	1.6200	0.9970	1.0700	1.2200	1.0700	1.0800
4-6-1-0-1	0.0494	0.0450	0.0950	0.0584	0.0476	0.0523	0.0119	0.0200	0.0401	0.0313	0.0419	0.0295
4-5-0-0-2	0.0165	0.0244	0.0191	0.0169	0.0168	0.0163	0.0042	0.0107	0.0344	0.0195	0.0232	0.0208
4-5-2-0-0	5.9000	5.4200	4.0900	6.2400	6.3100	5.2100	2.4300	5.7100	5.2400	5.6200	4.7600	5.5800
7-7-0-3-0	0.1920	0.0627	0.1140	0.2400	0.1130	0.3420	0.0737	0.1220	0.1530	0.1180	0.0652	0.2240
7-7-1-2-0	0.0642	0.0363	0.0111	0.0306	0.0479	0.0531	0.0802	0.0554	0.0307	0.0208	0.0338	0.0384
3-3-0-0-0	0.5010	0.4700	0.3870	0.6290	0.8450	0.3510	0.4180	0.7510	0.4820	0.5470	0.5760	0.5400
3-4-1-1-0	0.1000	0.1100	0.0451	0.0789	0.0799	0.0707	0.0196	0.0673	0.0875	0.0924	0.1230	0.0685
6-6-0-0-0	0.8560	0.8280	1.0100	0.8220	1.2200	1.1700	4.0400	0.9130	0.6220	0.9300	0.8980	0.8600
4-7-1-0-1	0.2250	0.2810	0.2390	0.2870	0.2100	0.1940	0.0293	0.1650	0.2540	0.1850	0.2020	0.1790
5-4-1-0-1	0.0995	0.1210	0.1020	0.1160	0.1060	0.1030	0.0518	0.1040	0.1960	0.1350	0.1130	0.1410
5-5-0-1-0	0.0994	0.1210	0.1020	0.1160	0.1060	0.1020	0.0506	0.1040	0.1940	0.1350	0.1130	0.1410
8-9-0-3-0	0.0042	0.0018	0.0041	0.0045	0.0044	0.0047	0.0011	0.0024	0.0051	0.0036	0.0063	0.0040
5-6-1-0-0	0.0181	0.0192	0.0209	0.0244	0.0148	0.0185	0.0104	0.0171	0.0355	0.0307	0.0162	0.0245
6-7-1-4-0	0.0014	0.0006	0.0015	0.0014	0.0008	0.0012	0.0007	0.0003	0.0017	0.0015	0.0018	0.0013
2-6-0-0-0	0.9630	0.9640	0.5410	0.8660	0.6560	0.7610	0.4620	0.5930	0.5800	0.5350	0.4610	0.4990

N-Glycan	CM_1	CM_2	CM_3	CM_4	CM_5	CM_6	CF_1	CF_2	CF_3	CF_4	CF_5	CF_6
3-4-0-1-0	0.7500	0.6140	0.5000	0.6400	0.6630	0.7770	0.2280	0.5280	0.7980	0.7240	0.7920	0.6470
3-4-1-0-1	0.8380	0.8830	0.6110	0.8240	0.8260	0.9990	0.3460	0.7660	0.9660	0.7860	0.7560	0.8770
4-7-1-0-0	0.0133	0.0216	0.0262	0.0161	0.0158	0.0101	0.0096	0.0174	0.0289	0.0180	0.0193	0.0144
7-5-0-0-0	0.2770	0.2400	0.2650	0.1460	0.3520	0.1050	1.0000	0.7430	0.1350	0.1490	0.2800	0.1420
7-5-0-1-0	0.0156	0.0081	0.0495	0.0025	0.0238	0.0151	0.1270	0.0682	0.0063	0.0082	0.0247	0.0044
2-5-0-0-0	1.6700	1.3100	1.0200	1.4900	1.1200	1.3200	1.6400	1.2900	1.0700	1.0800	0.9220	1.1400
4-4-1-0-0	4.2600	4.7700	4.7900	3.7200	3.0900	2.6700	3.8800	4.4800	5.7000	4.4800	4.8400	3.7400
5-5-1-1-0	0.0185	0.0190	0.0085	0.0271	0.0144	0.0287	0.0047	0.0050	0.0169	0.0079	0.0216	0.0312
3-4-0-0-1	0.6760	0.7310	1.0200	0.6160	0.6870	0.8170	0.5300	0.5770	0.7340	0.7650	1.0700	0.6710
5-5-0-0-2	1.3300	0.7040	2.0100	0.8880	1.1600	2.4300	4.4300	1.1900	0.7910	1.2200	1.3900	1.3500
6-5-0-0-0	0.0259	0.0111	0.0122	0.0102	0.0326	0.0033	0.0023	0.0129	0.0083	0.0074	0.0220	0.0121
4-6-1-0-0	0.5560	0.6860	0.6120	0.6640	0.5660	0.5250	0.2820	0.5560	0.7970	0.5830	0.6310	0.5490
5-6-0-0-3	0.1160	0.1020	0.1790	0.1070	0.0792	0.1800	0.0110	0.1060	0.1580	0.1600	0.1490	0.2300
5-4-0-0-0	0.1180	0.1360	0.2420	0.1220	0.1310	0.1280	0.3780	0.1220	0.2520	0.2120	0.1970	0.2200
7-6-1-0-1	0.4280	0.2670	0.5880	0.6290	0.6900	0.7870	0.6440	0.3340	0.3780	0.3910	0.4610	0.5000
3-5-1-0-0	0.1360	0.0866	0.0809	0.0833	0.1010	0.1040	0.0362	0.0701	0.1320	0.1000	0.1390	0.0862
7-5-1-1-0	0.0619	0.0828	0.0359	0.0283	0.0908	0.0104	0.0886	0.2010	0.0225	0.0392	0.1460	0.0626
3-5-0-0-1	0.0518	0.0528	0.0346	0.0258	0.0439	0.0193	0.1020	0.0489	0.0107	0.0134	0.0429	0.0132
4-3-0-0-0	0.2590	0.3130	0.3060	0.2440	0.2120	0.1630	0.3130	0.3350	0.3300	0.3440	0.4090	0.2880
4-4-1-0-1	1.4700	1.3900	1.3600	1.6300	1.2800	1.1800	0.5740	1.5200	2.0400	1.5100	1.5800	1.4900
5-8-1-0-1	0.2140	0.2240	0.2600	0.1260	0.1980	0.0976	0.3720	0.1620	0.0926	0.1320	0.1470	0.1140
4-4-0-0-1	0.1300	0.1340	0.1870	0.1030	0.1070	0.1500	0.0936	0.0781	0.1590	0.1520	0.1480	0.1490
4-5-0-0-0	0.0061	0.0066	0.0039	0.0049	0.0061	0.0027	0.0037	0.0035	0.0037	0.0045	0.0083	0.0039
4-5-0-1-0	1.4800	1.3900	1.4100	1.6300	1.2800	1.1800	0.6150	1.5200	2.0400	1.5300	1.5800	1.4900
5-6-0-0-1	0.0269	0.0218	0.0585	0.0208	0.0593	0.0882	0.1700	0.0556	0.0346	0.0718	0.0881	0.0498
6-5-1-1-0	0.3280	0.2410	0.4230	0.2580	0.4500	0.5770	2.7000	0.3430	0.3300	0.6000	0.2090	0.8180
6-7-0-4-0	0.0148	0.0062	0.0123	0.0136	0.0131	0.0092	0.0055	0.0052	0.0145	0.0081	0.0146	0.0095
7-5-1-0-0	0.2120	0.2940	0.0955	0.1090	0.3070	0.0419	0.4400	0.5750	0.0663	0.1070	0.3260	0.1430
5-7-1-2-0	0.7440	0.4530	0.7780	0.8350	0.6650	0.7550	0.2500	0.3280	0.5760	0.5560	0.6910	0.6020
8-7-0-0-1	0.0540	0.0551	0.0342	0.0928	0.0713	0.0519	0.0016	0.0243	0.0337	0.0305	0.0559	0.0674
4-4-0-1-0	1.1500	0.9740	0.8370	1.0800	0.9160	0.9500	0.2980	0.9470	0.8470	0.8530	0.9700	0.7890
5-3-0-0-0	0.1200	0.1320	0.0774	0.1260	0.1200	0.1300	0.1240	0.2320	0.1320	0.1510	0.1300	0.1460
5-5-1-0-1	0.0668	0.0857	0.0728	0.0688	0.0460	0.0658	0.1130	0.0643	0.0795	0.0764	0.0590	0.0675
5-8-2-0-0	0.9440	1.0300	0.8230	1.0900	0.9050	1.1600	0.4530	0.8590	1.1100	1.0300	0.8730	1.0500
7-7-0-1-0	0.8400	0.4950	0.8430	0.8560	0.9510	1.0500	0.8390	0.4770	0.5230	0.6700	0.7490	0.7310
4-3-1-0-0	24.200	27.200	23.300	24.100	24.300	17.700	26.100	31.500	26.200	25.300	26.600	24.300
6-4-1-1-0	0.0574	0.0361	0.0108	0.0419	0.0628	0.0175	0.0071	0.0504	0.0171	0.0390	0.0331	0.0333
6-5-1-0-1	4.7200	3.9100	6.2100	4.5800	5.2700	8.4000	6.0100	4.1900	3.3600	5.0500	3.7700	5.2000
5-5-1-0-2	0.6390	0.6370	0.9240	0.4900	0.7870	1.3400	1.3400	1.0200	0.6250	0.9710	0.6880	1.0700
6-5-0-2-0	0.0590	0.0460	0.0801	0.0615	0.0759	0.1220	0.0176	0.0440	0.0695	0.1240	0.1080	0.1370
9-7-0-1-0	0.0037	0.0013	0.0027	0.0032	0.0026	0.0039	0.0003	0.0011	0.0029	0.0018	0.0037	0.0034
7-5-0-0-1	0.1370	0.1480	0.3100	0.0754	0.1630	0.0790	0.4140	0.1970	0.0653	0.1010	0.2220	0.1130
7-8-1-0-1	0.3270	0.1940	0.4130	0.3880	0.3560	0.5480	0.0412	0.2340	0.3710	0.3440	0.3890	0.3810
4-6-0-0-1	0.0140	0.0214	0.0124	0.0067	0.0026	0.0037	0.0641	0.0175	0.0143	0.0116	0.0150	0.0066
4-7-0-0-1	0.0589	0.1140	0.0149	0.0702	0.0666	0.0910	0.0137	0.0358	0.0419	0.0570	0.0631	0.0636

N-Glycan	CM_1	CM_2	CM_3	CM_4	CM_5	CM_6	CF_1	CF_2	CF_3	CF_4	CF_5	CF_6
6-6-0-2-0	0.7480	0.6200	1.0500	0.6420	0.7120	1.4400	1.0400	0.7350	0.5930	0.9060	0.6580	0.9120
2-4-0-0-0	0.0047	0.0045	0.0016	0.0019	0.0020	0.0029	0.0047	0.0023	0.0028	0.0026	0.0027	0.0014
5-6-0-3-0	2.9400	1.9300	2.1600	3.1100	2.7800	2.9900	0.7060	1.3400	2.2700	2.1300	2.6300	2.2200
5-6-1-3-0	0.6350	0.3980	0.6520	0.7020	0.6520	0.7320	0.0740	0.3160	0.5850	0.5460	0.6310	0.5910
4-12-0-0-0	0.0412	0.0163	0.4280	0.0257	0.0927	0.2460	0.0931	0.0376	0.0967	0.2380	0.0658	0.2840
4-5-1-0-2	1.0100	1.5200	0.9880	1.3600	1.1600	1.1000	0.1420	0.8740	1.4400	1.0300	1.1000	1.1200
4-7-0-0-2	0.0086	0.0242	0.0193	0.0092	0.0165	0.0132	0.0199	0.0167	0.0109	0.0067	0.0173	0.0108
5-6-0-4-0	1.3800	0.8650	1.4200	1.4400	1.3700	2.0400	0.1580	0.7650	1.4100	1.1800	1.1800	1.4300
6-5-1-2-0	0.0120	0.0136	0.0074	0.0159	0.0108	0.0191	0.0080	0.0092	0.0098	0.0072	0.0135	0.0247
6-7-0-3-0	0.0715	0.0394	0.1160	0.0763	0.0501	0.0671	0.0127	0.0447	0.0794	0.0689	0.0808	0.0818
7-5-1-0-2	0.0139	0.0059	0.0744	0.0103	0.0334	0.0437	0.0313	0.0081	0.0267	0.0551	0.0341	0.0713
6-6-1-2-0	0.1280	0.1140	0.2270	0.1090	0.1330	0.2310	0.2520	0.1650	0.1270	0.2070	0.1980	0.2550
8-6-1-0-2	0.0120	0.0048	0.0012	0.0166	0.0107	0.0035	0.0011	0.0042	0.0040	0.0026	0.0078	0.0069
3-6-1-0-1	0.0340	0.0424	0.0222	0.0375	0.0328	0.0231	0.0108	0.0261	0.0346	0.0311	0.0302	0.0263
5-6-0-2-0	0.5370	0.3820	0.5610	0.5670	0.3960	0.4950	0.1620	0.3510	0.5430	0.4020	0.6800	0.4460
5-7-0-0-1	1.6100	1.4000	2.8600	1.2300	1.7700	2.8100	4.7800	1.1500	0.8330	1.5000	1.6300	1.2300
7-4-1-0-1	0.7940	0.7860	0.6710	0.3430	0.9170	0.3150	1.2700	1.4000	0.2850	0.4370	0.9090	0.4220
7-5-0-0-2	0.0126	0.0031	0.1070	0.0103	0.0266	0.0381	0.0293	0.0083	0.0209	0.0457	0.0275	0.0410
8-6-0-2-0	0.0273	0.0169	0.0347	0.0120	0.0421	0.0112	0.0386	0.0367	0.0058	0.0075	0.0479	0.0138
5-4-1-0-0	0.4140	0.6230	0.4080	0.3810	0.3530	0.4300	0.5130	0.4220	0.6640	0.5590	0.4670	0.6100
6-7-0-0-4	0.2110	0.1550	0.3170	0.2370	0.2080	0.2460	0.1470	0.1160	0.1830	0.1800	0.2420	0.2200
5-7-1-0-1	0.0183	0.0222	0.0203	0.0134	0.0154	0.0175	0.0665	0.0059	0.0086	0.0155	0.0240	0.0145
8-5-1-0-1	0.0874	0.0667	0.0886	0.0558	0.1320	0.0370	0.1250	0.1260	0.0419	0.0486	0.1600	0.0593
8-6-0-1-0	0.0899	0.0694	0.0968	0.0594	0.1400	0.0385	0.1300	0.1350	0.0432	0.0520	0.1780	0.0624
5-5-0-2-0	0.0865	0.0727	0.0687	0.0929	0.0748	0.0955	0.0198	0.0739	0.0833	0.0680	0.1020	0.0773
5-5-1-2-0	0.1520	0.1250	0.6110	0.0957	0.1320	0.2530	0.0512	0.0817	0.1660	0.1550	0.1540	0.1780
6-6-1-0-0	2.3400	2.6000	3.7500	2.2100	2.9100	3.9500	8.1900	1.9300	2.2600	2.8700	2.7400	3.3400
7-8-1-0-0	1.7800	1.0700	1.6200	1.4800	1.9100	1.6200	1.0900	0.9580	1.0600	1.1800	1.5900	1.2100
5-3-1-0-0	6.8200	9.3900	7.0100	8.6400	7.8900	6.9800	9.4700	8.9300	10.600	9.2700	8.3000	10.400
7-5-0-2-0	0.0419	0.0590	0.0454	0.0135	0.0574	0.0201	0.0583	0.0954	0.0239	0.0365	0.0855	0.0448
10-7-0-0-0	0.0119	0.0103	0.0052	0.0056	0.0099	0.0029	0.0063	0.0137	0.0027	0.0054	0.0173	0.0044
Total relative abundance (%)	100.0079	100.0461	99.9853	99.9704	100.0181	99.9491	99.982	99.9697	100.0967	100.0393	100.0264	99.9939

Table S2. Percentage relative abundances of *N*-glycans derived from GBH-Exposed sample cohort. *N*-Glycan nomenclature as was described in the main manuscript. EM refers to Exposed Male, while EF refers to Exposed Female.

N-Glycan	EM_1	EM_2	EM_3	EM_4	EM_5	EF_1	EF_2	EF_3	EF_4	EF_5	EF_6	EF_7
4-5-1-1-0	0.0388	0.0516	0.0307	0.0479	0.0610	0.0474	0.0400	0.0479	0.0379	0.0382	0.0421	0.0513
4-5-1-2-0	0.9540	1.0400	1.0100	0.7850	1.0500	0.9670	1.0900	1.3900	0.9070	0.6250	1.2200	1.1900
4-5-0-2-0	4.8500	4.6900	5.1200	4.6400	5.7200	3.4600	4.7400	5.5000	2.9300	2.3200	4.0800	3.6200
4-6-1-2-0	0.3210	0.1950	0.3070	0.4270	0.2460	0.1920	0.2870	0.3330	0.2640	0.1620	0.3090	0.2810
3-4-0-0-0	0.0747	0.0630	0.0495	0.0769	0.0623	0.0688	0.0994	0.0582	0.0913	0.0362	0.0725	0.0793
4-4-1-1-0	0.0637	0.0704	0.0471	0.0701	0.0696	0.0539	0.0558	0.0568	0.0696	0.0345	0.0800	0.0798
6-6-1-1-0	0.0181	0.0358	0.0302	0.0333	0.0274	0.0473	0.0365	0.0508	0.0358	0.0421	0.0338	0.0393
3-6-1-0-0	0.0131	0.0092	0.0113	0.0118	0.0096	0.0064	0.0131	0.0086	0.0153	0.0041	0.0161	0.0128
4-5-1-0-1	0.1070	0.0992	0.0801	0.1130	0.1070	0.0826	0.0830	0.0896	0.0921	0.0485	0.0724	0.1010
4-6-0-1-0	0.1070	0.0986	0.0801	0.1130	0.1070	0.0826	0.0831	0.0895	0.0921	0.0514	0.0723	0.1020
3-3-1-0-0	0.6540	0.5550	0.7020	0.7700	0.6360	0.6070	0.5820	0.7220	0.8960	0.5630	0.7390	0.9470
4-5-3-0-1	2.9000	2.6800	2.6100	2.5100	2.5800	2.6700	2.4900	2.4200	2.9800	2.2300	3.4700	2.9900
4-7-0-1-0	0.1230	0.1200	0.1310	0.1340	0.1390	0.0836	0.1410	0.1420	0.0993	0.0440	0.1110	0.1140
4-6-1-1-0	5.5700	4.7700	6.4000	4.7700	5.1300	4.2900	4.5800	4.1100	4.3800	3.7200	4.8700	4.2200
6-6-0-1-0	0.0396	0.0395	0.0559	0.1170	0.0389	0.0865	0.0576	0.0774	0.0686	0.0681	0.0498	0.0543
3-4-1-0-0	0.0503	0.0615	0.0341	0.0700	0.0544	0.0575	0.0468	0.0470	0.0842	0.0623	0.0756	0.1030
3-5-0-1-0	0.7150	0.9540	0.8920	0.8540	0.8380	0.7630	0.9840	1.0900	0.9850	1.0400	1.0800	1.0400
4-5-0-0-1	0.1620	0.1320	0.1240	0.1950	0.1590	0.1040	0.1670	0.1340	0.1830	0.0720	0.1750	0.1790
5-6-1-2-0	0.0526	0.0646	0.0542	0.0382	0.0651	0.0509	0.0617	0.0558	0.0589	0.0327	0.0704	0.0599
7-5-1-0-1	0.0065	0.0033	0.0178	0.0079	0.0050	0.0231	0.0103	0.0235	0.0128	0.0053	0.0165	0.0120
3-6-0-1-0	0.6770	0.5680	0.7010	0.6770	0.5970	0.4970	0.6190	0.6010	0.6660	0.3800	0.6500	0.6510
3-5-1-0-1	1.4800	2.5600	2.3100	1.4100	2.0700	1.4100	1.5600	1.4700	0.9870	2.5000	1.2600	1.2400
4-6-1-0-1	0.0572	0.0634	0.0642	0.0640	0.0627	0.0418	0.0684	0.0596	0.0512	0.0177	0.0457	0.0541
4-5-0-0-2	0.0291	0.0270	0.0215	0.0390	0.0226	0.0145	0.0215	0.0223	0.0370	0.0029	0.0301	0.0358
4-5-2-0-0	6.8000	6.2200	6.6900	6.6300	6.3400	5.5500	5.6800	6.0600	5.4200	4.2600	6.5100	5.3700
7-7-0-3-0	0.1440	0.2620	0.2620	0.2170	0.2370	0.2500	0.2100	0.1540	0.1540	0.1410	0.1700	0.1620
7-7-1-2-0	0.0403	0.1140	0.0740	0.0172	0.0764	0.0614	0.0758	0.0639	0.0442	0.0662	0.0520	0.0494
3-3-0-0-0	0.5010	0.4100	0.2850	0.3550	0.4260	0.4330	0.7040	0.3760	0.4930	0.2850	0.4000	0.4870
3-4-1-1-0	0.0532	0.0585	0.0290	0.0608	0.0648	0.0468	0.0595	0.0498	0.0929	0.0320	0.0755	0.0908
6-6-0-0-0	0.9260	1.3700	1.2200	1.0600	1.3000	1.2600	1.1500	1.2000	0.9040	1.4800	0.8730	0.8730
4-7-1-0-1	0.2360	0.1650	0.1450	0.2000	0.1440	0.1340	0.1370	0.1450	0.2310	0.0847	0.1930	0.2030
5-4-1-0-1	0.1850	0.1250	0.0862	0.1450	0.1130	0.1390	0.1260	0.1610	0.1740	0.0747	0.1660	0.1560
5-5-0-1-0	0.1850	0.1250	0.0863	0.1450	0.1130	0.1390	0.1260	0.1600	0.1740	0.0756	0.1660	0.1560
8-9-0-3-0	0.0025	0.0067	0.0057	0.0015	0.0050	0.0041	0.0051	0.0075	0.0048	0.0032	0.0093	0.0060

N-Glycan	EM_1	EM_2	EM_3	EM_4	EM_5	EF_1	EF_2	EF_3	EF_4	EF_5	EF_6	EF_7
5-6-1-0-0	0.0351	0.0194	0.0184	0.0244	0.0201	0.0266	0.0247	0.0290	0.0287	0.0076	0.0333	0.0339
6-7-1-4-0	0.0007	0.0018	0.0018	0.0004	0.0018	0.0014	0.0020	0.0023	0.0008	0.0007	0.0029	0.0017
2-6-0-0-0	0.7060	0.4800	0.6600	0.6020	0.5830	0.4290	0.5370	0.3710	0.4860	0.2780	0.5290	0.5670
3-4-0-1-0	0.5590	0.5270	0.4490	0.7460	0.4660	0.3990	0.5620	0.6200	0.6860	0.3330	0.7230	0.6780
3-4-1-0-1	0.7530	0.6720	0.7970	0.7950	0.7660	0.5870	0.7930	0.7650	0.8330	0.7540	0.8000	0.8000
4-7-1-0-0	0.0304	0.0213	0.0212	0.0267	0.0191	0.0170	0.0158	0.0183	0.0286	0.0085	0.0220	0.0263
7-5-0-0-0	0.1580	0.3900	0.4310	0.4530	0.4370	0.5860	0.3510	0.5900	0.2850	0.2410	0.2330	0.1700
7-5-0-1-0	0.0044	0.0356	0.0507	0.0729	0.0422	0.0621	0.0413	0.1050	0.0263	0.0163	0.0157	0.0116
2-5-0-0-0	1.1300	1.0300	1.0400	1.2900	1.3000	0.9670	1.0600	1.4000	1.0800	0.5440	1.2000	1.0300
4-4-1-0-0	3.7600	3.3700	2.7400	3.6500	3.7400	3.6800	3.8000	3.7500	4.9800	2.9000	3.8800	4.5600
5-5-1-1-0	0.0234	0.0118	0.0316	0.0195	0.0153	0.0205	0.0245	0.0287	0.0277	0.0116	0.0255	0.0244
3-4-0-0-1	0.4300	0.7870	0.4120	0.5510	0.7680	0.5840	0.6450	0.5850	0.7610	1.2400	0.4850	0.5970
5-5-0-0-2	1.0900	2.5900	2.2900	1.9800	1.5800	1.6100	1.2900	1.4400	1.5200	5.0600	1.2200	0.9620
6-5-0-0-0	0.0023	0.0101	0.0198	0.0027	0.0107	0.0141	0.0110	0.0180	0.0023	0.0011	0.0080	0.0056
4-6-1-0-0	0.7190	0.5570	0.6120	0.6720	0.6870	0.5300	0.6780	0.6580	0.6600	0.3080	0.6620	0.6720
5-6-0-0-3	0.1130	0.0868	0.1560	0.2050	0.1170	0.1880	0.2030	0.1910	0.1360	0.1130	0.1840	0.1750
5-4-0-0-0	0.1150	0.1180	0.1060	0.1680	0.1280	0.1600	0.1830	0.2260	0.1960	0.0922	0.1810	0.1770
7-6-1-0-1	0.4290	0.5390	0.2140	0.2510	0.7160	0.5920	0.5020	0.3970	0.3520	0.3480	0.3530	0.3680
3-5-1-0-0	0.0754	0.0496	0.0683	0.1020	0.0473	0.0366	0.0948	0.0876	0.1080	0.0500	0.1180	0.0894
7-5-1-1-0	0.0115	0.0202	0.0840	0.0634	0.0201	0.1250	0.0259	0.1120	0.0517	0.0212	0.0374	0.0198
3-5-0-0-1	0.0196	0.0207	0.0257	0.0424	0.0336	0.0417	0.0224	0.0302	0.0199	0.0189	0.0180	0.0202
4-3-0-0-0	0.2850	0.1720	0.2380	0.6290	0.2120	0.2120	0.2250	0.1880	0.3120	0.1450	0.3410	0.4440
4-4-1-0-1	1.8200	1.3500	1.2900	1.5400	1.6000	1.1900	1.6400	1.6400	1.5600	0.9090	1.6700	1.6400
5-8-1-0-1	0.1200	0.1080	0.1150	0.1890	0.1520	0.2280	0.1250	0.1500	0.1180	0.1410	0.1100	0.1040
4-4-0-0-1	0.1070	0.1300	0.1050	0.1560	0.1300	0.1360	0.1260	0.1260	0.1270	0.0797	0.1190	0.1240
4-5-0-0-0	0.0022	0.0020	0.0037	0.0062	0.0035	0.0073	0.0047	0.0080	0.0019	0.0045	0.0037	0.0032
4-5-0-1-0	1.8200	1.3500	1.2900	1.5400	1.6000	1.1900	1.6400	1.6200	1.5600	0.9090	1.6700	1.6400
5-6-0-0-1	0.0267	0.1660	0.0762	0.0396	0.1010	0.0528	0.0778	0.1070	0.0644	0.2940	0.0509	0.0476
6-5-1-1-0	0.3790	0.3560	0.4390	0.4550	0.3690	0.5210	0.4890	0.4840	0.5250	0.6460	0.4560	0.4770
6-7-0-4-0	0.0080	0.0213	0.0148	0.0048	0.0163	0.0076	0.0167	0.0183	0.0075	0.0053	0.0186	0.0141
7-5-1-0-0	0.0450	0.0850	0.2060	0.1340	0.0974	0.2590	0.1550	0.1910	0.1390	0.1150	0.1120	0.0760
5-7-1-2-0	0.5410	0.5740	0.6240	0.3630	0.6500	0.4770	0.6570	0.6140	0.4710	0.3080	0.7010	0.6290
8-7-0-0-1	0.0528	0.0354	0.0704	0.0194	0.0370	0.0448	0.0507	0.0374	0.0169	0.0117	0.0440	0.0471
4-4-0-1-0	1.0800	0.8330	0.8420	1.0600	1.0200	0.5840	0.8580	0.7970	0.6190	0.3990	0.8870	0.9010
5-3-0-0-0	0.1080	0.2070	0.0798	0.1870	0.1600	0.1320	0.1050	0.1480	0.2120	0.0756	0.4000	0.1710
5-5-1-0-1	0.0834	0.0631	0.0545	0.0583	0.0587	0.0746	0.0576	0.0684	0.0774	0.0273	0.0811	0.0742
5-8-2-0-0	0.6730	0.8120	0.9760	1.0700	0.8560	0.7270	0.8590	1.0500	1.0200	0.9520	1.0600	0.9600
7-7-0-1-0	0.6320	0.8570	0.7500	0.3740	0.9060	0.8360	0.7650	0.7020	0.4850	0.8070	0.6220	0.6210
4-3-1-0-0	26.300	22.600	21.000	22.800	23.200	26.200	24.600	23.300	27.600	26.000	23.300	27.200
6-4-1-1-0	0.0286	0.0277	0.0475	0.0287	0.0299	0.0375	0.0306	0.0214	0.0236	0.0284	0.0361	0.0221
6-5-1-0-1	4.3200	6.1300	6.5800	6.9600	4.9700	5.4000	4.4400	4.6000	4.8300	9.0100	4.0800	4.0800
5-5-1-0-2	0.6920	1.2700	0.8920	0.7350	0.7870	1.1700	0.5890	0.9580	1.0700	2.4400	0.8070	0.7320
6-5-0-2-0	0.0462	0.0781	0.0859	0.0988	0.0650	0.1170	0.0858	0.1090	0.0472	0.0955	0.0838	0.0800
9-7-0-1-0	0.0021	0.0046	0.0030	0.0021	0.0037	0.0030	0.0036	0.0036	0.0024	0.0016	0.0036	0.0028
7-5-0-0-1	0.0560	0.0905	0.2050	0.2750	0.1200	0.1710	0.1420	0.1730	0.1350	0.1420	0.0857	0.0605

N-Glycan	EM_1	EM_2	EM_3	EM_4	EM_5	EF_1	EF_2	EF_3	EF_4	EF_5	EF_6	EF_7
7-8-1-0-1	0.3420	0.2740	0.3890	0.2290	0.2810	0.2320	0.3880	0.3790	0.3000	0.1610	0.4110	0.4090
4-6-0-0-1	0.0084	0.0078	0.0060	0.0132	0.0110	0.0217	0.0056	0.0245	0.0197	0.0032	0.0104	0.0122
4-7-0-0-1	0.0613	0.0586	0.0500	0.0735	0.0497	0.0275	0.0416	0.0355	0.0806	0.0129	0.0743	0.0643
6-6-0-2-0	0.6300	0.8570	0.9880	1.6300	0.6820	0.9820	0.7200	0.7650	0.8850	1.2500	0.6600	0.6460
2-4-0-0-0	0.0019	0.0016	0.0028	0.0036	0.0025	0.0022	0.0021	0.0031	0.0034	0.0009	0.0030	0.0024
5-6-0-3-0	2.5100	2.6000	2.5500	1.5300	2.9400	2.1100	2.6200	2.1500	1.7300	1.3200	2.9500	2.1800
5-6-1-3-0	0.5050	0.7000	0.5480	0.2720	0.7360	0.5270	0.5590	0.6340	0.4310	0.2750	0.7270	0.6290
4-12-0-0-0	0.0975	0.0346	0.0634	0.0395	0.0693	0.1640	0.1830	0.1420	0.1670	0.2340	0.1710	0.1540
4-5-1-0-2	1.5200	1.1600	0.8310	1.3300	0.9820	0.7680	0.8460	0.9770	1.2800	0.5890	1.2600	1.3800
4-7-0-0-2	0.0094	0.0098	0.0096	0.0135	0.0091	0.0166	0.0137	0.0147	0.0167	0.0224	0.0132	0.0133
5-6-0-4-0	1.2000	1.4100	1.8000	1.0100	1.3600	1.0600	1.6000	1.5500	0.9900	0.6310	1.5300	1.3800
6-5-1-2-0	0.0122	0.0109	0.0204	0.0107	0.0064	0.0195	0.0132	0.0056	0.0057	0.0123	0.0084	0.0142
6-7-0-3-0	0.0501	0.0831	0.0667	0.0542	0.0945	0.0581	0.0805	0.0795	0.0464	0.0295	0.0872	0.0749
7-5-1-0-2	0.0205	0.0151	0.0178	0.0095	0.0270	0.0515	0.0456	0.0446	0.0313	0.0381	0.0542	0.0462
6-6-1-2-0	0.0775	0.1640	0.1840	0.2140	0.1420	0.2640	0.1840	0.2030	0.1870	0.3100	0.1440	0.1570
8-6-1-0-2	0.0081	0.0071	0.0242	0.0027	0.0102	0.0042	0.0101	0.0066	0.0003	0.0016	0.0069	0.0042
3-6-1-0-1	0.0384	0.0368	0.0417	0.0333	0.0172	0.0309	0.0258	0.0228	0.0284	0.0234	0.0293	0.0377
5-6-0-2-0	0.4950	0.4700	0.4700	0.3030	0.6160	0.3980	0.5830	0.5020	0.3840	0.2520	0.5710	0.5550
5-7-0-0-1	1.0100	1.9100	2.3700	2.1200	1.8700	2.0300	1.6300	1.5200	1.2300	3.4600	1.0400	0.9590
7-4-1-0-1	0.2710	0.4530	0.9610	1.3700	0.5170	0.8900	0.5880	0.7500	0.6940	0.5790	0.4500	0.3340
7-5-0-0-2	0.0246	0.0111	0.0075	0.0002	0.0240	0.0410	0.0449	0.0262	0.0143	0.0582	0.0426	0.0215
8-6-0-2-0	0.0069	0.0241	0.0537	0.0190	0.0313	0.0421	0.0390	0.0681	0.0119	0.0058	0.0146	0.0118
5-4-1-0-0	0.4720	0.3680	0.3270	0.4940	0.4280	0.6200	0.5010	0.4950	0.5600	0.3330	0.4740	0.5720
6-7-0-0-4	0.1740	0.2440	0.2050	0.1490	0.2460	0.2240	0.2210	0.2360	0.1620	0.1460	0.2350	0.2280
5-7-1-0-1	0.0111	0.0177	0.0188	0.0236	0.0145	0.0318	0.0157	0.0169	0.0164	0.0325	0.0096	0.0101
8-5-1-0-1	0.0392	0.0694	0.1130	0.0731	0.0970	0.1360	0.1010	0.1630	0.0715	0.0136	0.0761	0.0479
8-6-0-1-0	0.0424	0.0713	0.1220	0.0813	0.0998	0.1490	0.1090	0.1810	0.0748	0.0162	0.0802	0.0516
5-5-0-2-0	0.0963	0.0826	0.0807	0.0658	0.1010	0.0639	0.0762	0.0752	0.0575	0.0375	0.0854	0.0966
5-5-1-2-0	0.1340	0.1010	0.1800	0.1800	0.1620	0.1360	0.1950	0.1460	0.1420	0.1290	0.1460	0.0785
6-6-1-0-0	2.2800	4.0600	3.2500	1.8100	3.2700	3.4400	3.0200	2.1500	2.2100	4.7200	2.5800	2.9700
7-8-1-0-0	1.1700	1.5200	1.6300	1.0400	1.6300	1.5600	1.5400	1.5700	1.0800	1.0900	1.4700	1.2900
5-3-1-0-0	9.1800	6.7100	6.5300	8.4100	6.0900	9.6600	9.1400	9.5000	10.000	7.7300	9.7700	9.6100
7-5-0-2-0	0.0079	0.0242	0.0928	0.1560	0.0343	0.0813	0.0443	0.0928	0.0583	0.0199	0.0437	0.0214
10-7-0-0-0	0.0055	0.0029	0.0120	0.0116	0.0032	0.0265	0.0117	0.0136	0.0088	0.0014	0.0061	0.0017
Total relative abundance (%)	100.0417	100.0370	99.9993	100.0232	99.9735	100.0276	99.9975	100.0571	99.9873	100.0345	99.9636	100.0128

Table S3: *N*-Glycans in the total rat group validated by LC-PRM-MS, including precursor m/z, transition fragment ions, fold change (FC) and log2FC for the full scan and PRM validation. *N*-Glycan nomenclature as was described in the main manuscript.

***N*-Glycan Composition**	**Precursor (m/z)**	**Transition ion fragments**	**FC (Full Sc)**	**Log2FC (Full Scan)**	**FC (PRM)**	**Log2FC (PRM)**
3-3-1-0-0	793.4283	564.3324,858.4355,899.4633,1117.5630,432.2139	0.75	-0.42	0.53	-0.92
3-4-0-0-0	808.4336	858.4355,464.2496,1103.5583,1172.0844,1271.8253	0.76	-0.40	0.62	-0.69
3-4-1-0-0	895.4782	344.1713,464.2494,1103.5571,858.4435,654.3315	0.75	-0.41	0.68	-0.56
3-5-1-0-1	1193.1202	1715.8447,1511.7659,825.4170,1266.6376 450.2334	1.33	0.41	1.08	0.11
3-6-0-1-0	795.7461	622.3079,825.4136,1048.5068,1293.6376, 1497.7585	0.91	-0.13	1.00	-0.01
3-6-1-0-0	733.3846	640.3135,858.4352,1089.5422,1293.6274,1508.1992	0.59	-0.76	0.98	-0.02
4-4-1-1-0	799.4181	746.6317,899.4576,1103,5470,1349.6877,1553.7693	0.75	-0.41	0.98	-0.04
4-5-0-0-1	819.4251	464.2502,674.4156,1089.5499,1293.6254,1538.7429	0.80	-0.32	0.96	-0.07
4-5-0-2-0	1394.2203	825.4249,1529.4758,1668.8064,1913.9435,1089.5632	1.68	0.75	1.06	0.09
4-5-1-2-0	1481.2649	825.4260,1089.5531,1668.8391,1913.9462,793.4042	1.59	0.67	1.13	0.18
4-6-1-0-1	1417.7332	751.3870,825.4235,855.432,1913.9775,1668.8333	1.24	0.32	1.05	0.08
4-6-1-1-0	935.4846	825.4250,1089.5471,1293.6405,1538.7504 1734.9041	0.84	-0.25	0.97	-0.05
4-6-1-2-0	1583.3148	825.4210,1186.5811,1405.6821,1668.8203,2276.1460	1.72	0.78	1.12	0.16
4-7-0-1-0	1417.7333	855.4417,1089.5402,1307.6603,1552.7756,1756.9005	1.36	0.44	1.02	0.03
5-6-1-2-0	1137.5846	607.3074,844.4182,1194.1896,1450.7083,1899.9628	1.32	0.40	1.13	0.17
7-5-1-0-1	1122.5811	825.4232,1350.6914,1122.7202,607.3077,1899.9479	0.60	-0.74	1.00	0.00
7-7-0-3-0	1073.8022	825.4211,1186.5921,1421.6889,1681.7393,2091.0564	1.30	0.38	1.03	0.05
7-7-1-2-0	1369.0354	825.4193,1176.8767,1334.4459,1695.8579,1960.3641	1.46	0.55	1.03	0.04

Table S4: *N*-Glycans in the rat gender subgroup validated by LC-PRM-MS, including precursor m/z, transition fragment ions, fold change (FC), and log2FC for the full scan and PRM validation. *N*-Glycan nomenclature as was described in the main manuscript.

***N*-Glycan Composition**	**Precursor (m/z)**	**Transition ion fragments**	**FC (Full Scan)**	**Log2FC (Full Scan)**	**FC (PRM)**	**Log2FC (PRM)**
3-4-0-0-0	808.4336	858.4355,464.2496,1103.5583,1172.0844,1271.8253	0.73	-0.46	0.84	-0.25
3-5-1-0-1	1193.1202	1715.8447,1511.7659,825.4170,1266.6376,450.2334	1.45	0.53	1.19	0.25
4-4-1-1-0	799.4180	746.6317,899.4576,1103,5470,1349.6877,1553.7693	0.71	-0.50	0.96	-0.06
4-5-0-0-1	819.4251	464.2502,674.4156,1089.5499,1293.6254,1538.7429,	0.77	-0.37	0.99	-0.02
4-5-1-0-1	1315.6833	855.4417,1089.5402,1307.6603,1552.7756,1756.9005	1.65	0.72	1.01	0.02
4-5-3-0-1	993.5143	825.4272,1089.5398,1293.6456,1538.7562,1914.0178	0.78	-0.36	0.96	-0.06
4-6-0-1-0	1315.6833	855.4417,1089.5402,1307.6603,1552.7756,1756.9005	1.67	0.74	1.03	0.05
4-6-1-2-0	1583.3148	825.4210,1186.5811,1405.6821,1668.8203,2276.1460	1.80	0.84	1.06	0.08
4-7-1-0-1	1013.5214	855.4365,1089.5427,1334.6688,1538.7634,1944.9298	0.74	-0.43	0.98	-0.03
6-6-1-1-0	1647.8542	607.3101,825.4267,998.4102,1304.2797,1668.8035	1.76	0.82	1.03	0.04
7-5-1-0-1	1122.5811	825.4232,1350.6914,1122.7202,607.3077,1899.9479	0.49	-1.03	0.89	-0.16
7-7-0-3-0*	1073.8021	825.4211,1186.5921,1421.6889,1681.7393,2091.0564	1.41	0.49	1.06	0.09

**N-glycan in Female gender subgroup*

Figure S1: *N*-glycan identification process: (**a**) Extracted Ion Chromatogram (EIC) of sialylated *N*-glycan, $HexNAc_4$, Hex_6, NeuAc (4-6-0-1-0) with the red and blue overlayed chromatogram extracted from control and GBH-exposed samples respectively. The inset provides the full MS spectrum of the *N*-glycan structure. (**b**) The MS/MS spectrum of the 4-6-0-1-0 with fragment ions labeled next to their corresponding peaks. *N*-Glycan symbols and nomenclature as described in **Figure 1**.

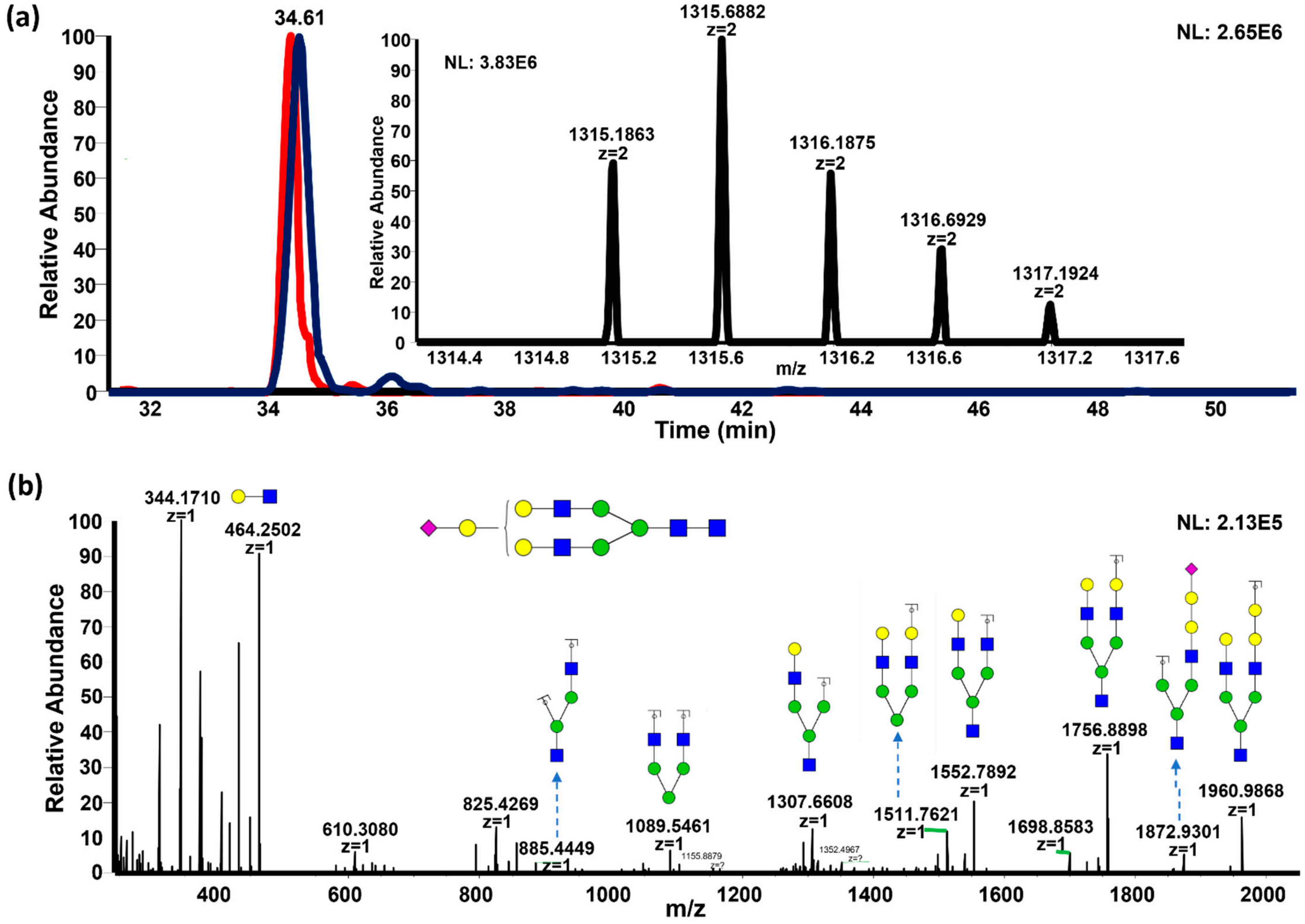

Figure S2. Distribution of all identified *N*-glycan by types between control *vs.* GBH-exposed cohorts in (**a**) total, (**b**) male, and (**c**) female gender subgroups. Statistical significance was performed using Mann–Whitney U test (* < 0.05)

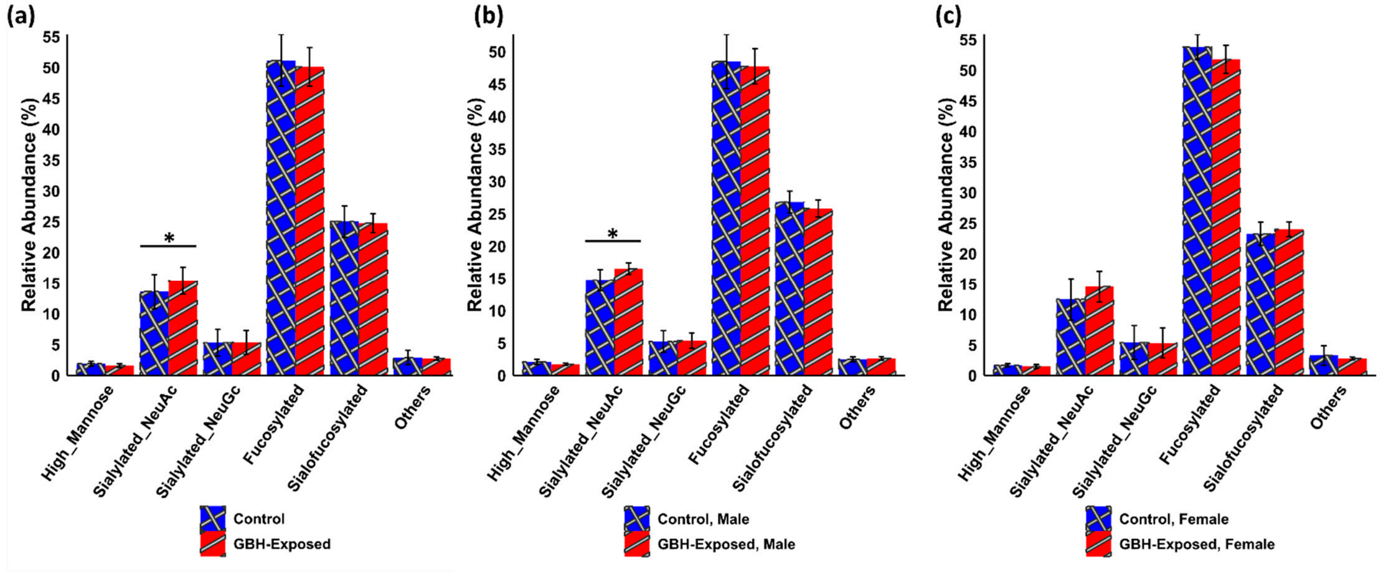

Figure S3. Venn plot shows unique and overlapping significant *N*-glycans in the total and gender subgroup data sets of comparison between control and GBH-exposed rats. *N*-Glycan nomenclature as described in **Figure 1**. *N*-Glycan symbols and nomenclature as described in **Figure 1**.

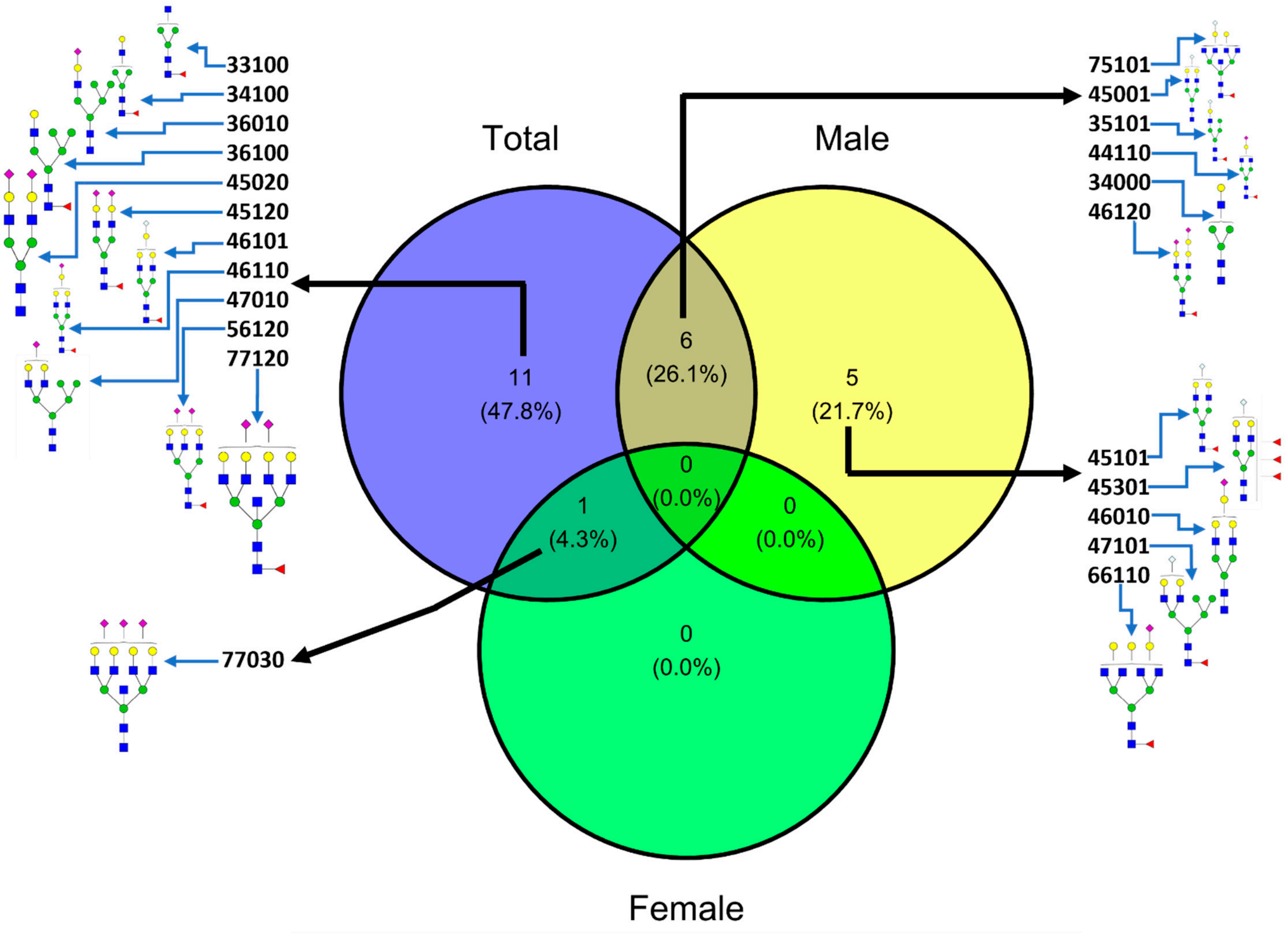

Figure S4: Box plots of the unique significant *N*-glycans: (**a**) Unique *N*-glycans in the total group, and (**b**) unique *N*-glycans in the male group. Statistical significance was performed using Mann–Whitney U test followed by BH correction procedure (* < 0.05, ** < 0.01). *N*-Glycan symbols and nomenclature as described in **Figure 1**.

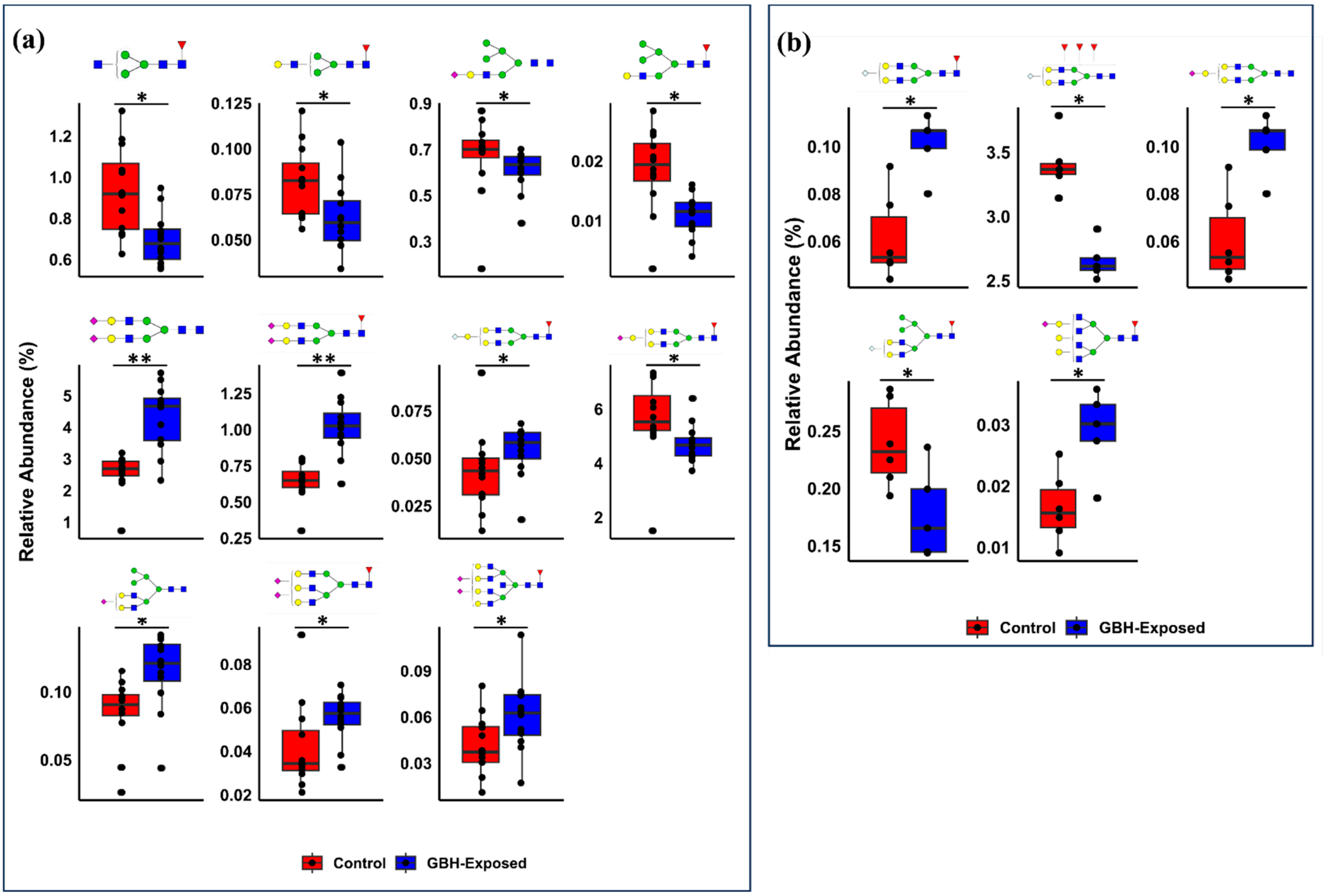

Supplement: Supplementary file 1 [file biomolecules-14-01077-s001.zip › biomolecules-3117365-supplementary.pdf]
